# Supplementary material for: Dopey1-Mon2 complex binds to dual-lipids and recruits kinesin-1 for membrane trafficking
Source: Nat Commun. 2019 Jul 19;10:3218. doi: 10.1038/s41467-019-11056-5 (PMC6642134; doi:10.1038/s41467-019-11056-5)
Supplement: Supplementary file 1 — Supplementary Information [file 41467_2019_11056_MOESM1_ESM.pdf]

Supplementary information

Dopey1-Mon2 complex binds to dual-lipids and recruits kinesin-1 for membrane trafficking

Mahajan et al.

Supplementary Figure 1

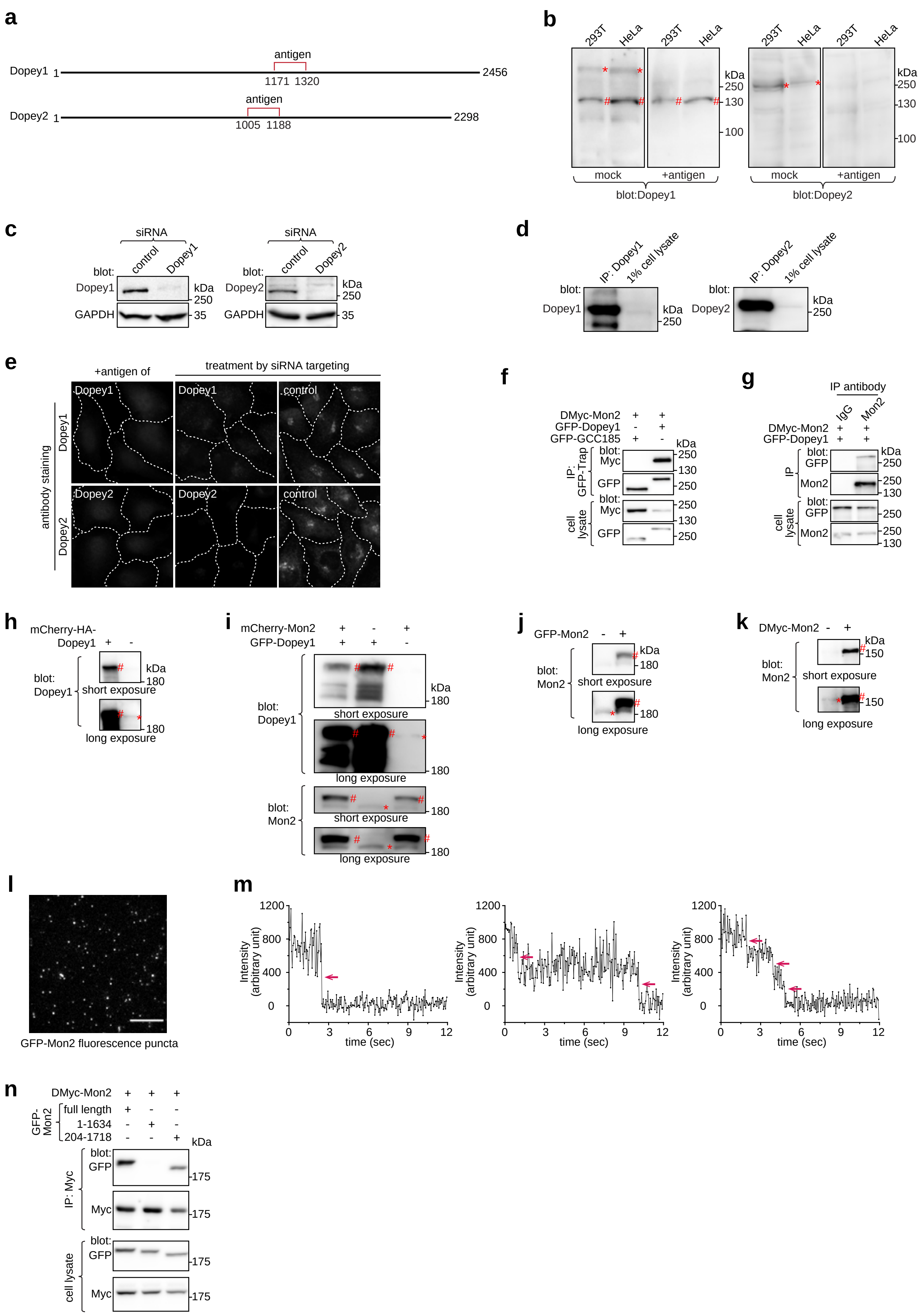

Supplementary Figure 1 Antibody characterization, single-molecule photo-bleaching experiment and interaction and expression of exogenously expressed Dopey1 and Mon2. **a** Schematic diagram showing regions of antigens used for Dopey1 and 2 antibodies. **b**, **c** Dopey1 and 2 antibodies can specifically detect endogenous corresponding proteins. In HeLa and HEK293T cell lysates, endogenous Dopey1 and 2 bands (indicated by ∗) can be detected by their antibodies. These bands disappeared if antibodies were pre-incubated with corresponding antigens (**b**) or when endogenous proteins were depleted by corresponding siRNAs in HeLa cells (**c**). #; non-specific band. **d** Dopey1 and 2 antibodies can efficiently IP endogenous corresponding proteins. Cell lysates were subjected to IP using indicated antibodies followed by immunoblotting using the same antibodies. **e** The immunofluorescence staining patterns of Dopey1 and 2 antibodies are specific. HeLa cells were used. The perinuclear staining patterns disappeared when antibodies were neutralized by corresponding antigens or when endogenous proteins were depleted by corresponding siRNAs. Scale bar, 10 µm. Dotted white lines indicate cell contours. **f**, **g** Exogenously expressed Dopey1 and Mon2 interact with each other. IPs were similarly performed as in Fig. 1b except that GFP-Trap agarose beads were used in **f**. **h**-**k** The exogenously expressed Dopey1 and Mon2 are quantitatively dominant over endogenous ones. Cell lysates expressing tagged Dopey1 or Mon2 were blotted with Dopey1 or Mon2 antibody. The overexpressed and endogenous protein levels can be compared in short and long exposure blot images. ∗ and # indicate endogenous and overexpressed corresponding proteins, respectively. **l** GFP-Mon2 molecules immobilized on a glass coverslip were imaged under TIRF microscopy. Scale bar, 10 µm. **m** Typical traces with 1, 2 and 3 single-molecule photo-bleaching steps. Arrows indicate photo-bleaching steps. **n** MEC is essential for the self-interaction of Mon2. Cell lysates co-expressing DMyc-Mon2 together with indicated truncations of GFP-Mon2 were subjected to IP followed by immunoblotting using indicated antibodies. MEC is partially deleted in GFP-Mon2(1-1634). Molecular weights (in kDa) are labeled in all gel blots. All cell lysates were from HEK293T unless specified otherwise. Source data are provided as a Source Data file.

Supplementary Figure 2

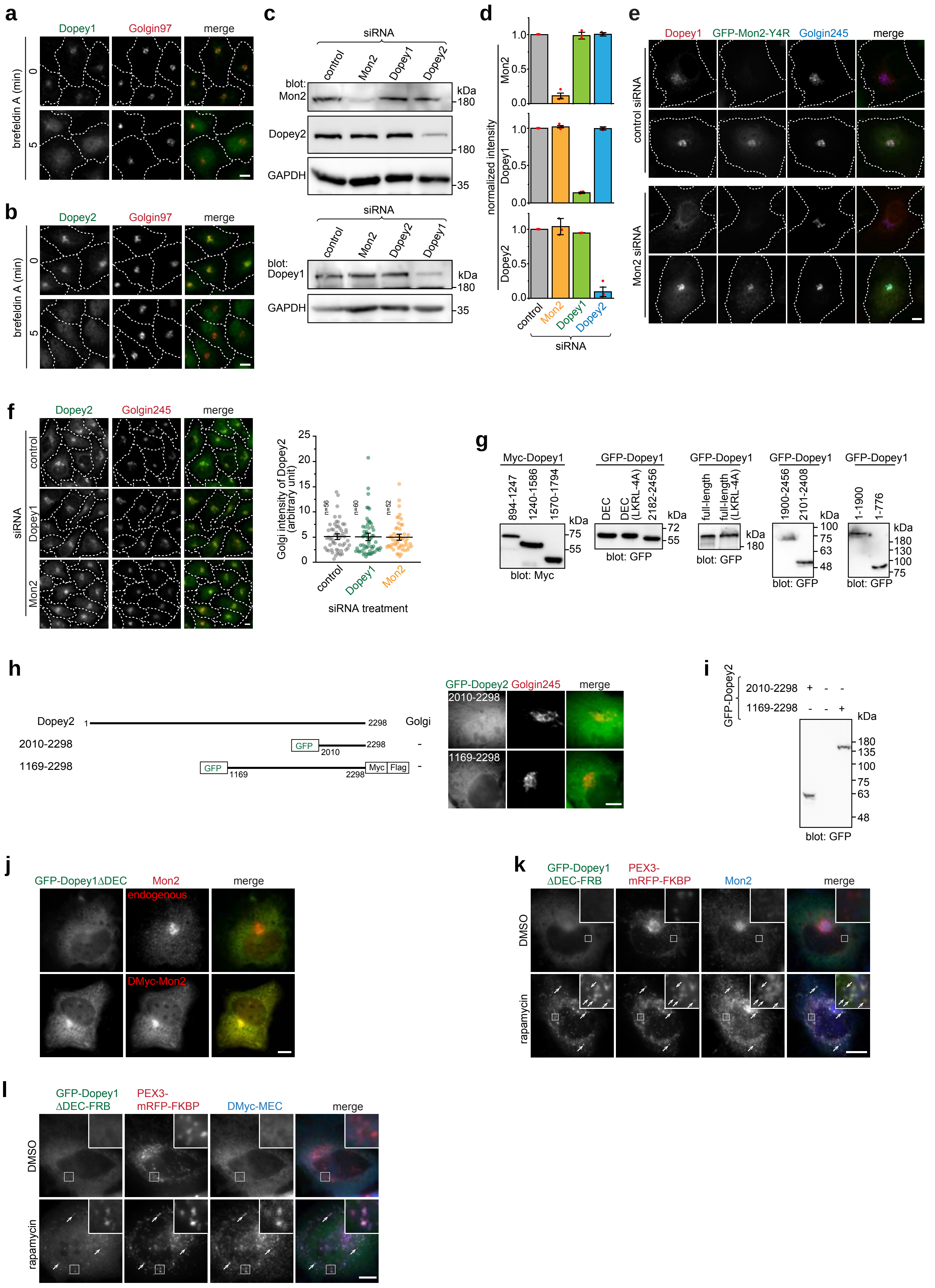

Supplementary Figure 2 The membrane localization of Dopey1 and its truncation mutants. All cells are HeLa cells. **a**, **b** Dopey1 and 2 are sensitive to brefeldin A treatment. Cells were treated with 10 µg ml^{-1} brefeldin A for 0 and 5 min before immunofluorescence co-staining of Dopey1 or 2 together with Golgin97 (a Golgi marker). **c**-**d** The depletion of Dopey1 or Mon2 did not affect the cellular expression level of Mon2 or Dopey1, respectively. The cellular level of Dopey2 was not affected either. Cell lysates subjected to knockdown by indicated siRNAs were immunoblotted by indicated antibodies. Band intensity was quantified and normalized by that of the corresponding GAPDH in **d**. Red dot, individual data point; error bar, mean ± s.d. from 3 independent experiments. **e** The loss of Dopey1's Golgi localization upon depletion of Mon2 can be rescued by the expression of an RNAi-resistant Mon2 construct. After Mon2 siRNA-mediated knockdown, cells were transfected to express the RNAi-resistant Mon2 construct, GFP-Mon2-Y4R, followed by immunofluorescence labeling of endogenous Dopey1 and Golgin245. **f** The Golgi localization of Dopey2 does not require Dopey1 or Mon2. The knockdown was conducted using corresponding siRNAs and cells were immunofluorescence labeled for Dopey2 and Golgin245. The Golgi fluorescence intensity of Dopey2 per cell was quantified at right. Error bar, mean ± s.e.m.; n, the number of cells analyzed. The result represents 3 independent experiments. **g** Dopey1 truncation or mutant constructs expressed corresponding proteins at expected sizes. Cell lysates expressing indicated constructs were blotted by indicated antibodies. **h** The C-terminal region of Dopey2 corresponding to DEC does not localize to the Golgi. The schematic diagram of truncation constructs (left panel) and their cellular localization (right panel). The Golgi localization results were obtained by imaging cells that expressed indicated constructs and stained for Golgin245. **i** Cell lysates expressing indicated constructs, which are described in **h**, were blotted by anti-GFP to demonstrate that they can express proteins of expected sizes. **j** Overexpressed Mon2 can recruit Dopey1∆DEC to the Golgi. Cells expressing GFP-Dopey1∆DEC alone (top row) or together with DMyc-Mon2 (bottom row) were subjected to immunofluorescence labeling of endogenous Mon2 or overexpressed DMyc-Mon2 using anti-Mon2 or anti-Myc antibody, respectively. **k**, **l** Peroxisome-tethered Dopey1 can recruit endogenous Mon2 or overexpressed MEC. Cells co-expressing indicated constructs were treated with 50 nM rapamycin or the same amount of DMSO solvent for 20 min before immunofluorescence labeling using anti-Mon2 (**k**) or anti-Myc antibody (**l**). The region of interest is enlarged in the upper right corner. Arrows demonstrate colocalization. Scale bar, 10 µm. Dotted white lines indicate cell contours (**a**, **b**, **e**, **f**). Molecular weights (in kDa) are labeled in all immunoblots. Source data are provided as a Source Data file.

Supplementary Figure 3

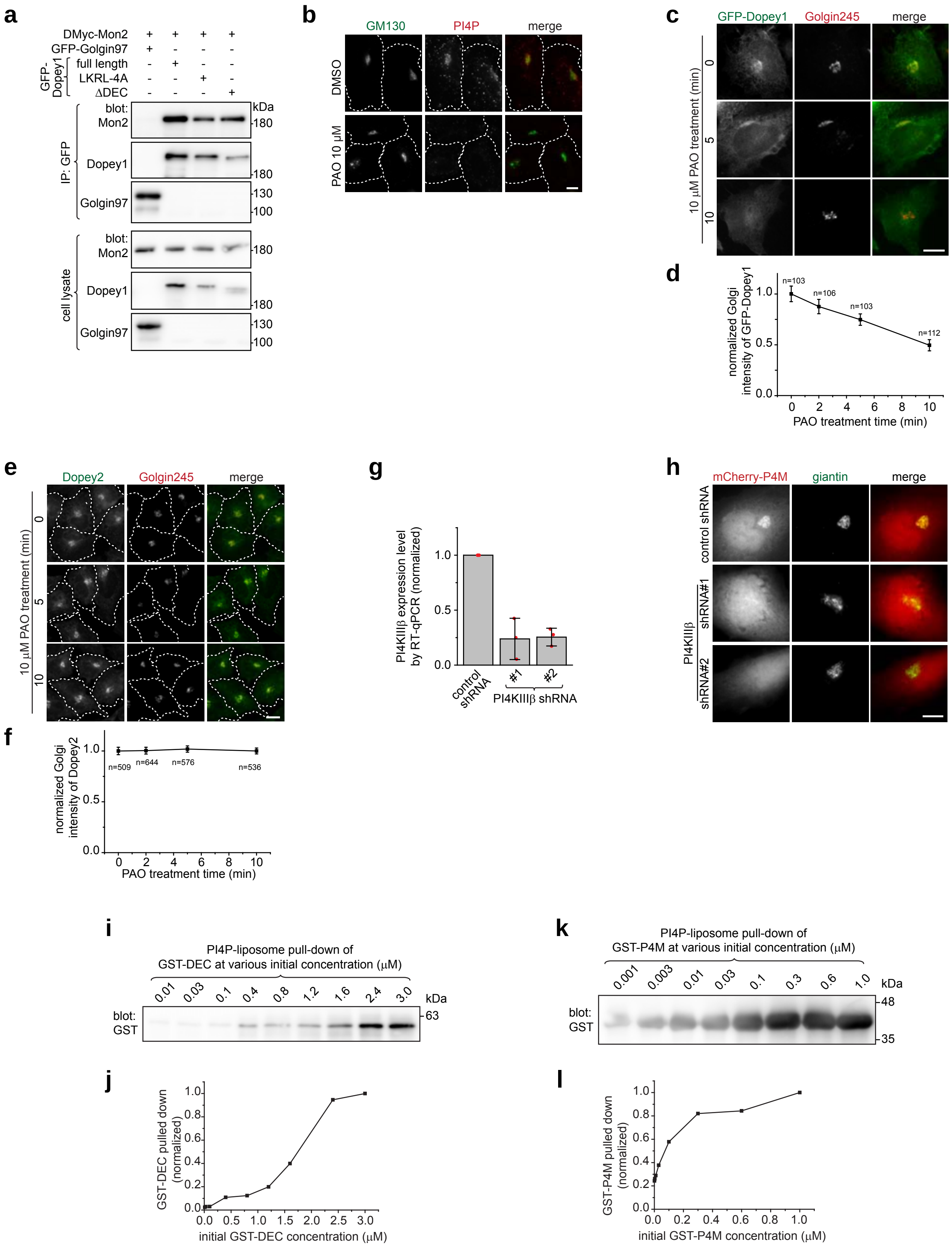

Supplementary Figure 3

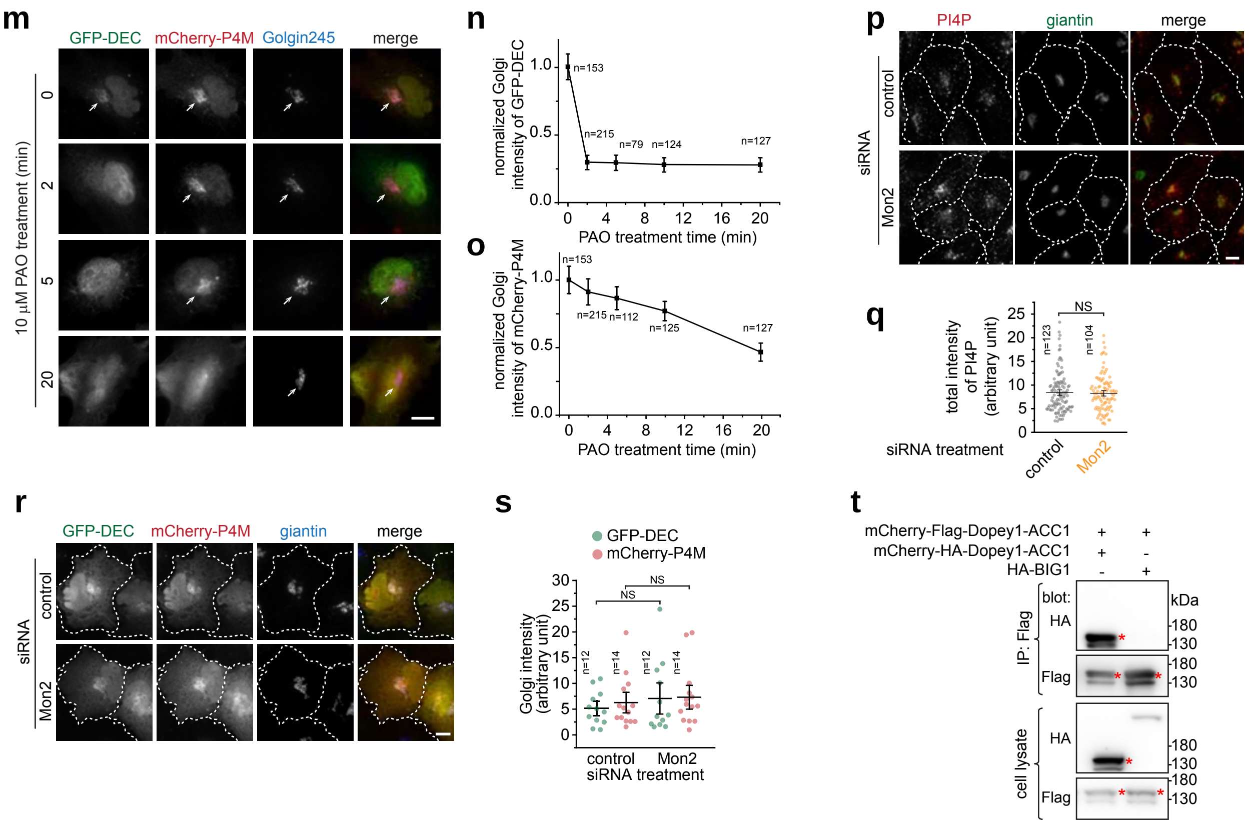

Supplementary Figure 3 DEC specifically interacts with PI4P. **a** DEC is not required for Dopey1's interaction with Mon2. IPs were similarly performed as in Fig. 1b. **b** PAO treatment depletes endogenous PI4P. Cells were treated with 10 μM PAO or equivalent DMSO for 10 min before immunofluorescence labeling of endogenous GM130 (a Golgi marker) and PI4P. **c**, **d** The Golgi localization of GFP-Dopey1 is sensitive to PAO. Cells transiently expressing GFP-Dopey1 were treated with 10 μM PAO and stained for endogenous Golgin245 in **c**. The normalized Golgi intensity per cell was quantified and plotted against time in **d**. **e**, **f** The Golgi localization of Dopey2 does not require PI4P. Cells were treated with 10 μM PAO and endogenous Dopey2 and Golgin245 were stained in **e**. The plot in **f** is similarly acquired as in **d**. **g** RT-qPCR demonstrated that PI4KIIIβ shRNA #1 and #2 can efficiently knockdown the transcript level of PI4KIIIβ. Red dot, individual data point; error bar, mean ± s.d. from 3 independent experiments. **h** The Golgi PI4P reduces upon PI4KIIIβ depletion. PI4KIIIβ knockdown cells were transiently transfected to express mCherry-P4M and processed for staining of giantin (a Golgi marker). **i-l** Estimating the PI4P binding affinity of DEC and P4M. Liposomes containing 1% (m/m) PI4P were incubated with serial dilutions of indicated recombinant GST-fusion proteins and the protein pulled down was quantified by immunoblotting. **m-o** DEC dissociates from the Golgi faster than P4M. Cells transiently co-expressing GFP-DEC and mCherry-P4M were treated with 10 μM PAO and endogenous Golgin245 was stained in **m**. The Golgi localization is indicated by arrows. Plots in **n**,**o** are similarly acquired as in **d**. **p-s** Mon2 depletion does not affect the intracellular level of PI4P and the Golgi association of DEC. After treatment with control or Mon2 siRNA, cells were either directly stained for endogenous PI4P and giantin in **p** or transfected to co-express indicated fusion proteins before immunostaining of giantin in **r**. Total intensity of PI4P per cell is quantified in **q** while the Golgi intensity of DEC and P4M per cell are quantified in **s**. **t** Dopey1-ACC1 dimerizes. IPs were similarly performed as in Fig. 1b. ∗, specific band. Molecular weights (in kDa) are labeled in all immunoblots. In **d**, **f**, **n**, **o**, **q** and **s**, error bar represents mean ± s.e.m and n is the number of cell analyzed. Scale bar, 10 μm. Dotted white lines indicate cell contours (**b**, **e**, **p**, **r**). **d**, **f**, **j**, **l**, **n**, **o**, **q**, **s** are representative results of 3 independent experiments. HEK293T cells were used in **a**,**t** while HeLa cells were used in **b-h** and **m-s**. Source data are provided as a Source Data file.

Supplementary Figure 4

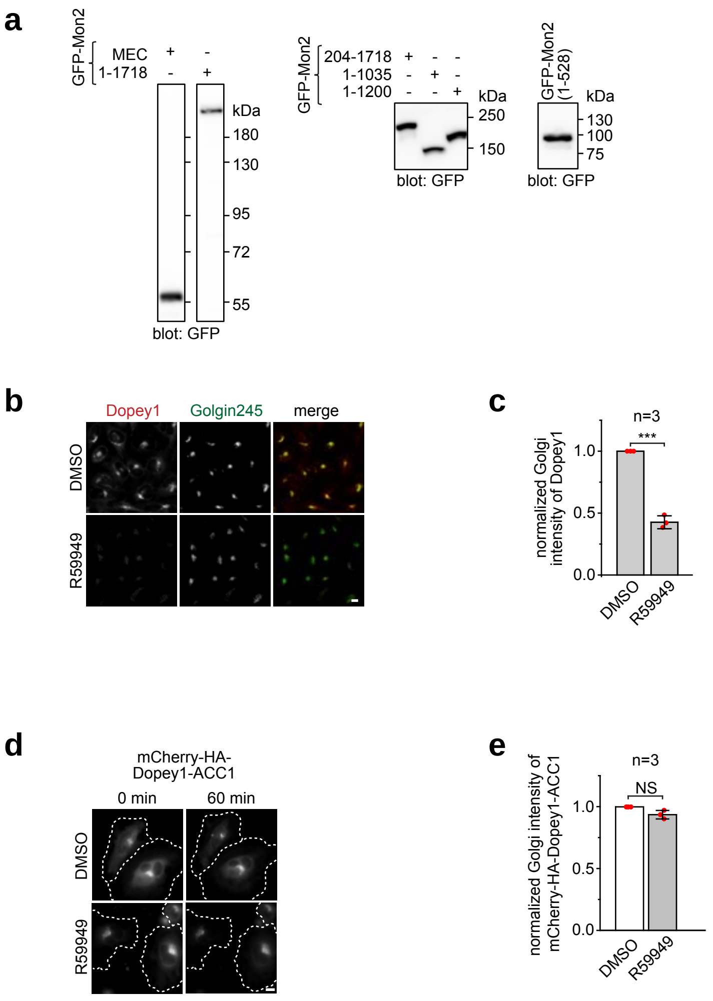

Supplementary Figure 4 The expression of Mon2 truncation constructs and the sensitivity of Dopey1's Golgi localization to PA-depletion. **a** HEK293T cell lysates expressing indicated constructs were blotted to demonstrate that Mon2 truncation constructs can express proteins of expected size. Molecular weights (in kDa) are labeled in all immunoblots. **b**, **c** The Golgi localization of Dopey1 is sensitive to PA-depletion. HeLa cells were treated with DMSO (control) or 1 µM R59949 for 1 h before immunostaining of endogenous Dopey1 and Golgin245. Normalized Golgi intensity of Dopey1 is plotted in **c**. Red dot, individual data point; error bar, mean ± s.d. from 3 independent experiments. **d**, **e** The Golgi localization of dimerized Dopey1 is insensitive to PA-depletion. HeLa cells transiently expressing mCherry-HA-Dopey1-ACC1 were treated with DMSO (control) or 1 µM R59949 for 1 h during live cell imaging. Dotted white lines indicate cell contours. In **e**, normalized Golgi intensity of Dopey1-ACC1 was calculated as the Golgi intensity after the treatment divided by that before the treatment. Red dot, individual data point; error bar, mean ± s.d. from 3 independent experiments. Scale bar, 10 µm. *P* values are from *t* test (unpaired and two-tailed); N.S., not significant; ∗∗∗, $P \leq 0.0005$. Source data are provided as a Source Data file.

Supplementary Figure 5

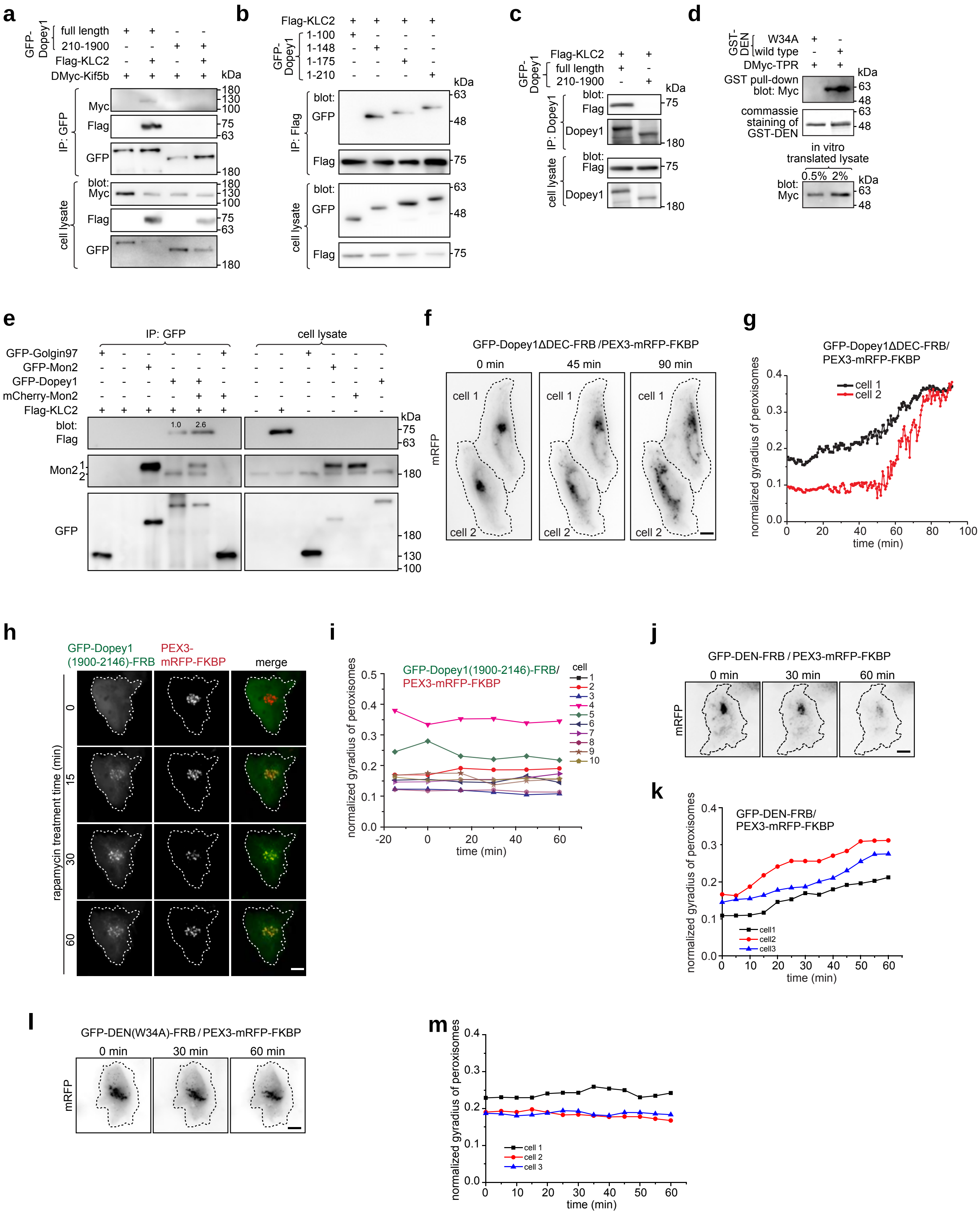

Supplementary Figure 5 DEN recruits kinesin-1 by directly interacting with KLC2. **a** Dopey1 indirectly interacts with Kif5b through KLC2. **b** Dopey1(1-148) is sufficient to interact with KLC2. This is the reverse IP experiment of Fig. 5b. **c** The Dopey1 fragment missing DEN does not interact with KLC2. In **a**-**c**, IPs were similarly performed as in Fig. 1b. **d** The TPR domain of KLC2 directly interacts with DEN. Indicated proteins were in vitro translated and mixed and the mixture was subjected to pull-down using bead-immobilized GST-DEN. **e** The interaction between Dopey1 and KLC2 is enhanced by Mon2. Cell lysates singly expressing tagged fusion proteins were mixed as indicated. Non-transfected cell lysate was used to balance the volume. Mixtures were subsequently subjected to IPs followed by immunoblotting using indicated antibodies. 1 and 2 indicate FP-tagged and endogenous Mon2, respectively. Values above Flag-KLC2 bands are normalized ratios of Flag-KLC2 band intensities to those of corresponding GFP-Dopey1 in the IP panel. **f**, **g** Live cell imaging of peroxisomes with artificially tethered Dopey1. Cells transiently expressing indicated constructs were treated with 50 nM rapamycin and imaged live. Selected peroxisome images (PEX3-mRFP-FKBP) from the time lapse are shown in **f**. The intensity of images is inverted. See also Supplementary movie 1. In **g**, the normalized gyradius of peroxisomes was quantified and plotted against the time. **h**, **i** Artificially tethering a C-terminal fragment of Dopey1 does not disperse peroxisomes. Time lapse images of a typical cell are shown in **h** and the normalized gyradius quantification results of 10 cells are shown in **i**. The experiment was similar to that in **f**, **g**. **j**-**m** Artificially tethering DEN, but not DEN(W34A), is sufficient to disperse peroxisomes from the cell center to the periphery. The figure layout and experiment are similar to **f**, **g**. See also Supplementary movie 2 for **j**. Scale bar, 10 µm. Cell contours are marked by dotted lines (**f**, **h**, **j**, **l**). Molecular weights (in kDa) are labeled in all immunoblots. HEK293T cells were used in **a**-**e** while HeLa cells were used in **f**-**m**. Source data are provided as a Source Data file.

Supplementary Figure 6

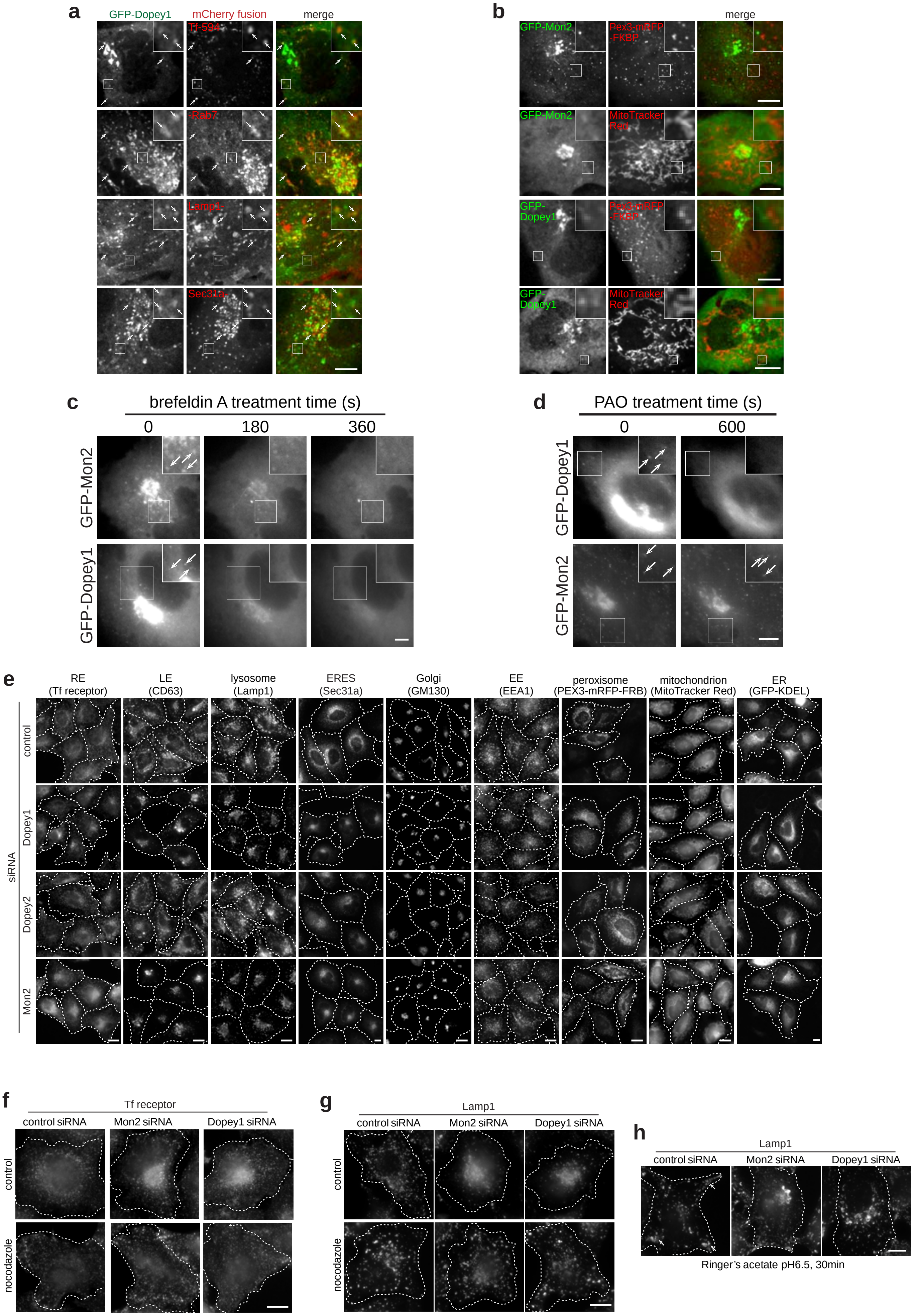

Supplementary Figure 6

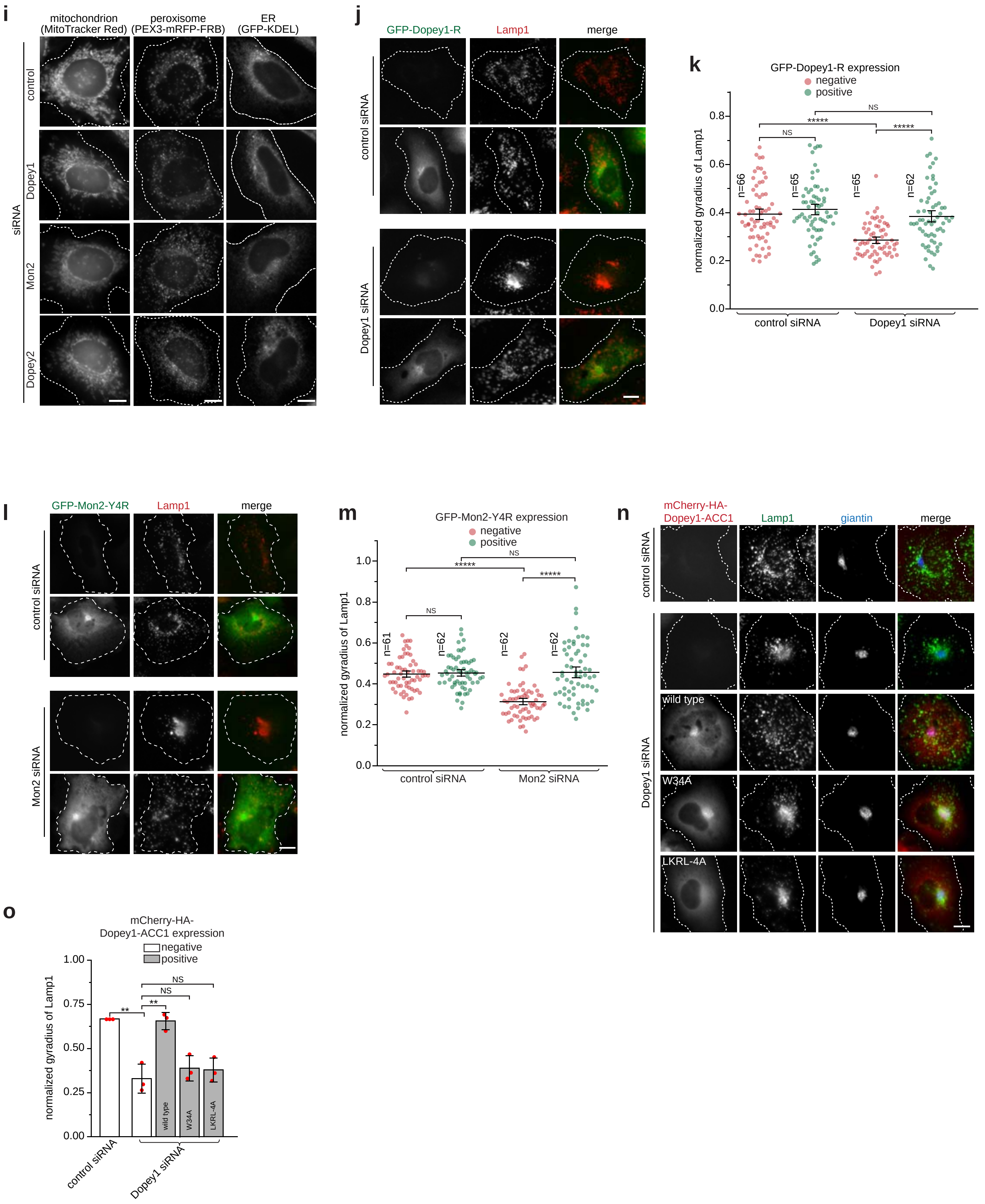

Supplementary Figure 6 Control experiments demonstrating that Dopey1-Mon2 complex is essential for the peripheral positioning of secretory and endocytic organelles. HeLa cells were used. **a** GFP-Dopey1 localizes to the EE/RE (Tf-594), LE (Rab7), lysosome (Lamp1) and ERES/ERGIC (Sec31a) in live cell imaging. The experiment and image organization are similar to Fig. 6a. **b** Dopey1 and Mon2 don't localize to the peroxisome and mitochondrion in live cell imaging. The experiment are similar to Fig. 6a. **c** The punctate (arrows) as well as the Golgi localization pattern of GFP-Mon2 and GFP-Dopey1 disappeared under brefeldin A treatment during live cell imaging. **d** The punctate (arrows) as well as the Golgi localization pattern of GFP-Dopey1, but not GFP-Mon2, disappeared under PAO treatment during live cell imaging. Regions of interest are enlarged at the upper right corner (**c**,**d**). **e** The effect of Dopey1 or Mon2 depletion on organelle positioning in a field of multiple cells. The experiment is similar to Fig. 6c,d. **f**, **g** Lysosomal aggregation induced by Dopey1 or Mon2 depletion requires microtubule network. After siRNA-mediated knockdown, cells were subjected to DMSO (control) or 33 µM nocodazole treatment for 30 min before immunofluorescence labeling of endogenous Lamp1 and Tf receptor. **h** Lysosomal aggregation induced by Dopey1 or Mon2 depletion is not reversed by pH 6.5 treatment. After the siRNA-mediated knockdown, cells were treated with Ringer's acetate solution (pH 6.5) for 30 min before immunofluorescence labeling of endogenous Lamp1. The periphery positioning of lysosomes induced by Ringer's acetate was demonstrated by the periphery accumulation of lysosomes (arrows) in the control panel. **i** The depletion of Dopey1 or Mon2 does not change the positioning of the mitochondrion, peroxisome and ER. After the siRNA-mediated knockdown, cells were transfected to express indicated marker constructs or stained by MitoTracker Red followed by fluorescence imaging. **j**, **k** Lysosomal aggregation induced by Dopey1 siRNA can be rescued by the expression of an RNAi-resistant Dopey1 construct. After the siRNA-mediated knockdown, cells were transfected to express the RNAi-resistant Dopey1 construct, GFP-Dopey1-R, followed by immunofluorescence labeling of endogenous Lamp1. The normalized gyradius of Lamp1 is quantified in **k**. **l**, **m** Lysosomal aggregation induced by Mon2 siRNA can be rescued by the expression of an RNAi-resistant Mon2 construct. The experiment and figure organization are similar to **j**, **k**. **k**, **m** are representative results from 3 independent experiments. n, the number of cells analyzed; error bar, mean ± s.e.m.. **n**, **o** Dopey1-ACC1 wild type, but not W34A and LKRL-4A mutant, can rescue Dopey1-depletion-induced lysosomal aggregation. The experiment and figure organization are similar to **j**, **k**. In **o**, red dot represents individual data point; error bar indicates mean ± s.d. from 3 independent experiments. *P* values are from *t* test (unpaired and two-tailed). N.S., not significant; **, $P \leq 0.005$; *****, $P \leq 0.000005$. Scale bar, 10 µm. Cell contours are marked by dotted white lines (**e**-**j**, **l, n**). Source data are provided as a Source Data file.

Supplementary Figure 7

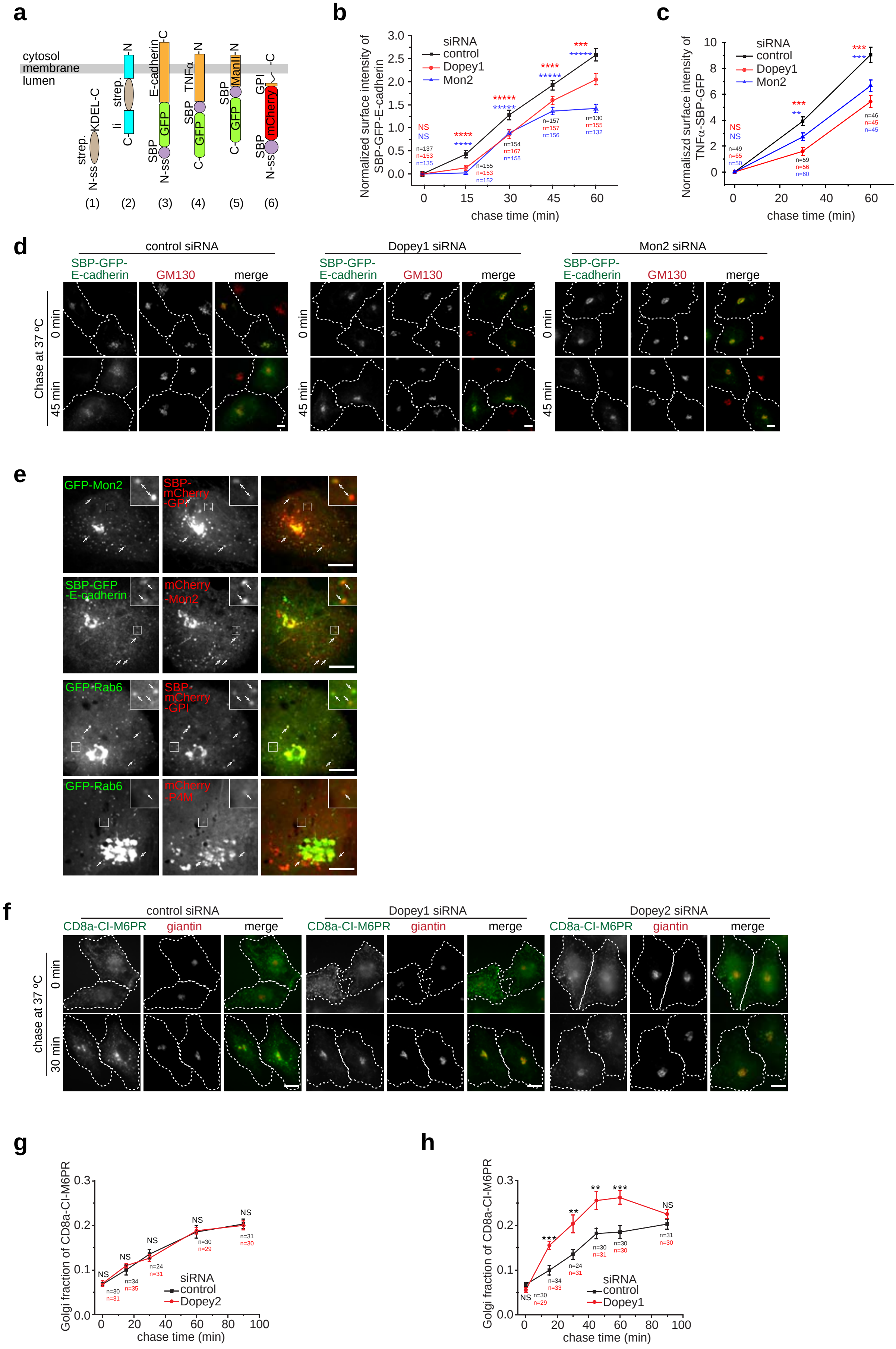

Supplementary Figure 7 Dopey1-Mon2 complex promotes the Golgi-to-PM but inhibits the reverse trafficking. All cells are HeLa cells. **a** Schematic diagram of RUSH hooks and reporters used in this study. N, N-terminus; C, C-terminus; strep., streptavidin; ss, signal sequence; SBP, streptavidin binding protein. (1) strep-KDEL (luminal hook), (2) strep-Ii (luminal hook), (3) SBP-GFP-E-cadherin (reporter), (4) TNFα-SBP-GFP (reporter), (5) ManII-SBP-GFP (reporter), (6) SBP-mCherry-GPI (reporter). **b**, **c** Depletion of Dopey1 or Mon2 inhibits the Golgi-to-PM trafficking of SBP-GFP-E-cadherin and TNFα-SBP-GFP. The experiment is similar to Fig. 7a. Normalized surface intensities of reporters, calculated as the surface GFP antibody intensity divided by the total cellular GFP intensity, are plotted against the chase time. **d** Depletion of Dopey1 or Mon2 inhibits the Golgi exit of SBP-GFP-E-cadherin. The experiment was similar to Fig. 7a. **e** Mon2, Rab6 and P4M can be found on membrane carriers involved in the Golgi-to-PM trafficking. The experiment and image organization are similar to Fig. 7d. **f-h** Depletion of Dopey1, but not Dopey2, accelerates the PM-to-Golgi trafficking. The experiment was similar to Fig. 7k,l except that siRNA #2 of Dopey1 and 2 were used. Scale bar, 10 μm; error bar, mean ± s.e.m. Dotted white lines indicate cell contours. In **b**, **c**, **g**, **h**, plots are representative results of 3 independent experiments; n indicates the number of cells analyzed. *P* values are from the *t* test (unpaired and two-tailed) comparison between Dopey1, Dopey2 or Mon2 and the corresponding control knockdowns (**b**, **c**, **g**, **h**). N.S., not significant. **, $P \leq 0.005$; ***, $P \leq 0.0005$; ****, $P \leq 0.00005$; *****, $P \leq 0.000005$. Source data are provided as a Source Data file.

Supplementary Figure 8

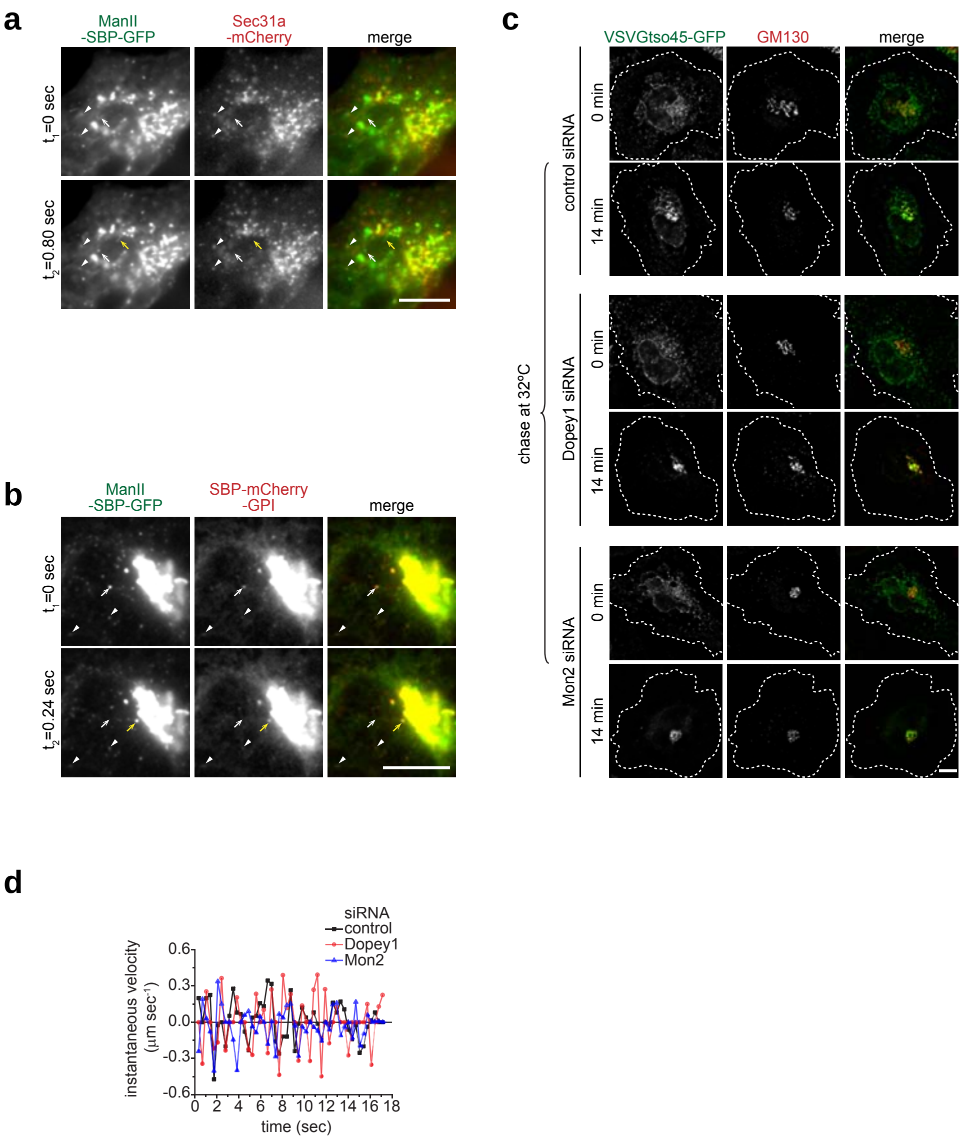

Supplementary Figure 8 Dopey1-Mon2 complex inhibits the ER-to-Golgi membrane trafficking. HeLa cells were used. **a** When released from the ER, the RUSH reporter ManII-SBP-GFP was first concentrated at Sec31a-positive and immobile ERES/ERGIC (arrow heads) and subsequently transported in rapid moving carriers (arrows). The experimental procedure and figure organization are similar to Fig. 8d,e. **b** When released from the ER, RUSH reporters, ManII-SBP-GFP and SBP-mCherry-GPI, colocalized at the same immobile puncta (arrow heads) and were transported in the same mobile carriers (arrow). The experimental procedure and figure organization are similar to Fig. 7d,e. **c** Depletion of Dopey1 or Mon2 promoted the ER-to-Golgi membrane trafficking of VSVGtso45-GFP. The experiment is the same as that of Fig. 8c. Representative deconvolved images are shown. Dotted white lines indicate cell contours. **d** The instantaneous velocity of a typical membrane carrier containing ManII-SBP-GFP in the ER-to-Golgi trafficking. The experiment is the same as those in Fig. 8f-j. Scale bar, 10 μm. Source data are provided as a Source Data file.

Supplementary Figure 9

Fig. 1a

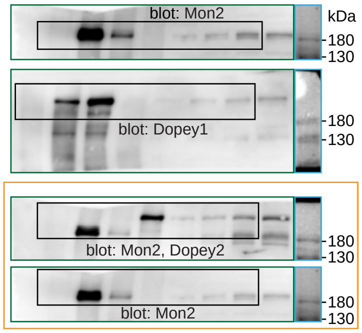

Fig. 1b

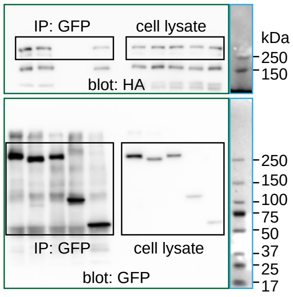

Fig. 1c

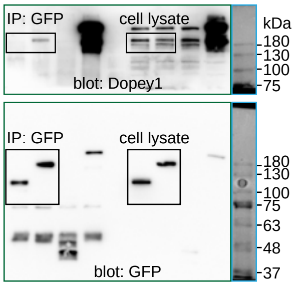

Fig. 1d

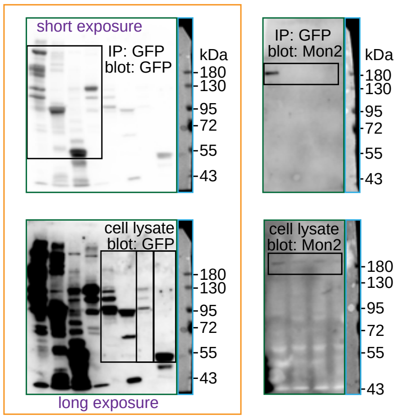

Fig. 1e

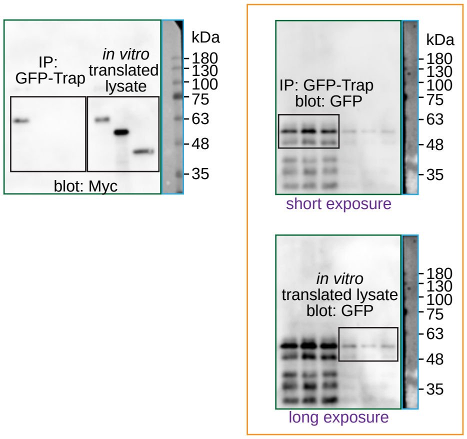

Fig. 1f

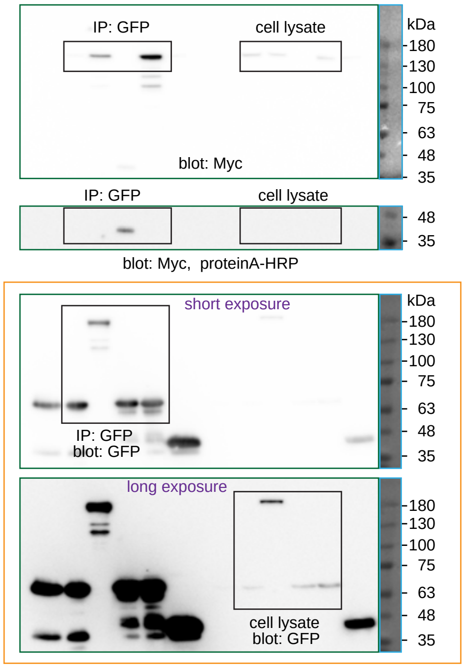

Fig. 1h

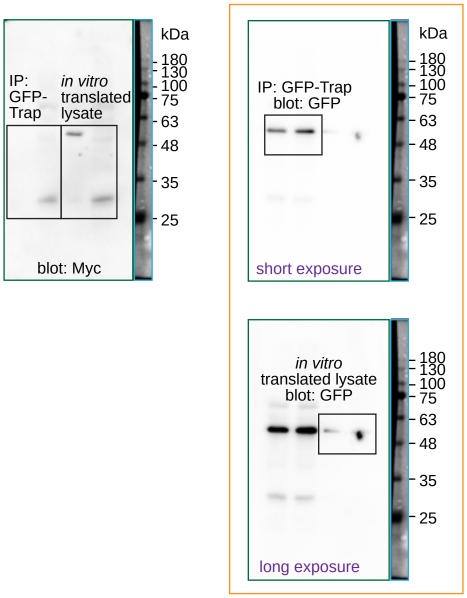

Supplementary Figure 9

Fig. 1i

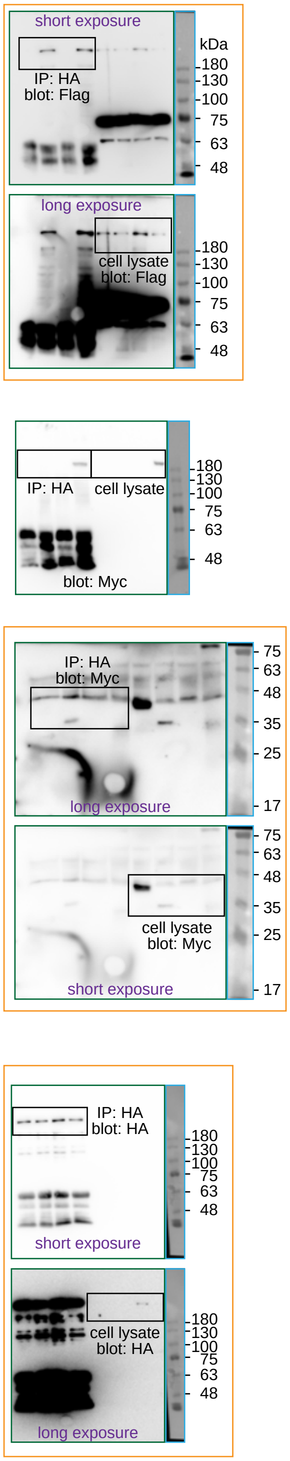

Fig. 3i

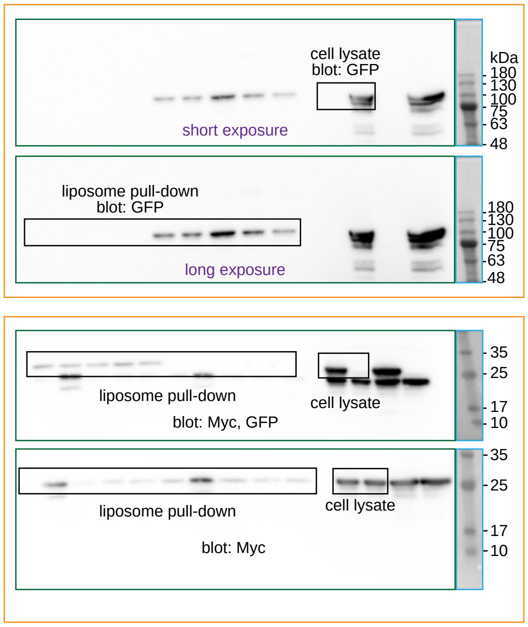

Fig. 3j

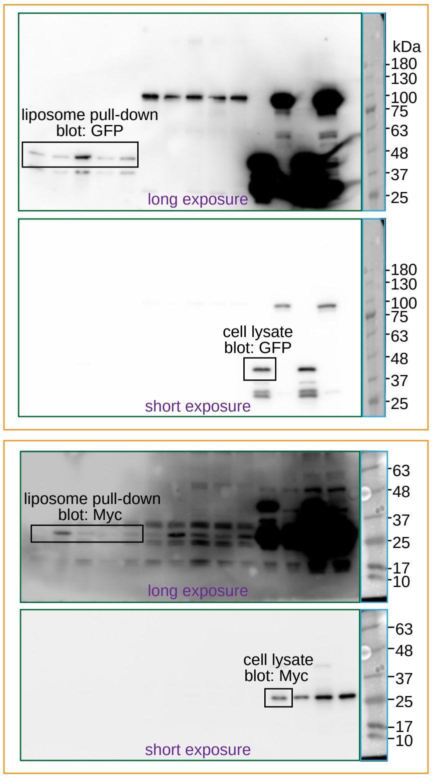

Fig. 3k

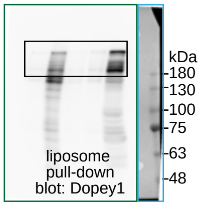

Fig. 3l

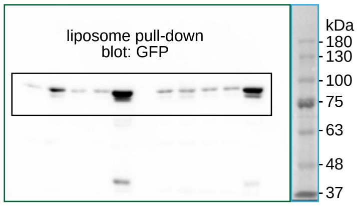

Fig. 3m

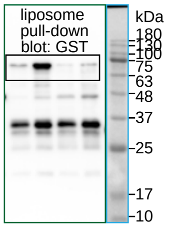

Supplementary Figure 9

Fig. 4e

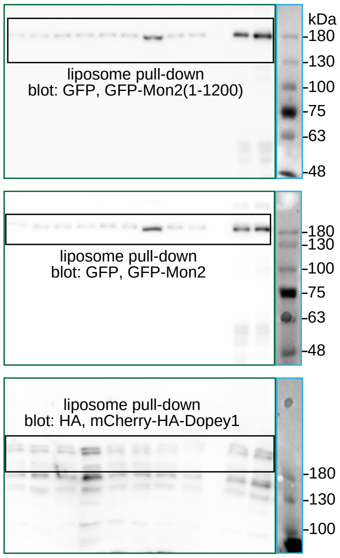

Fig. 4f

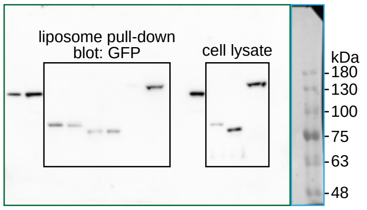

Fig. 5a

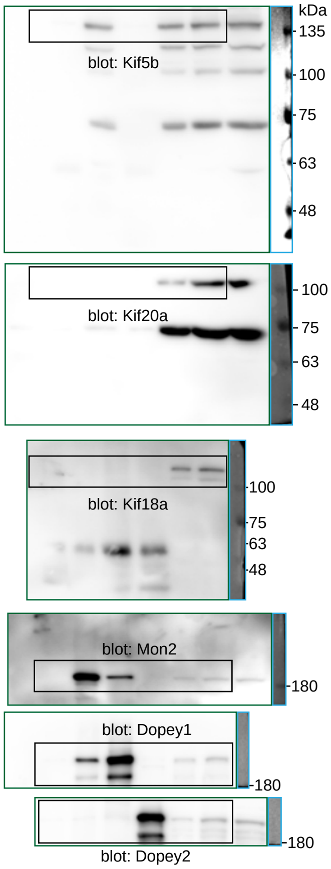

Fig. 5b

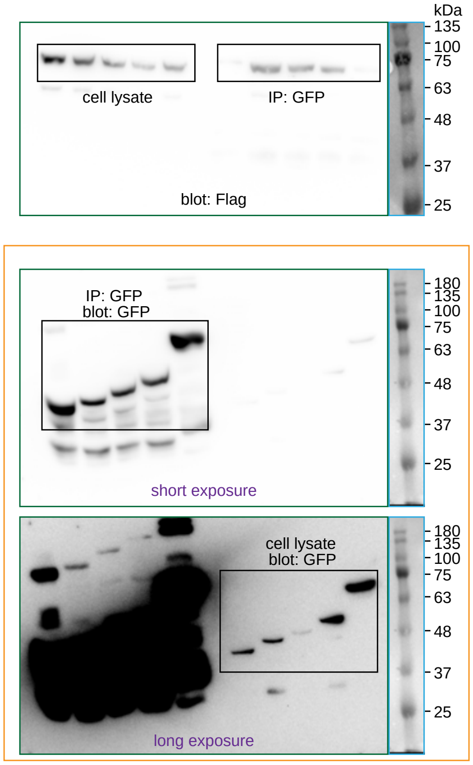

Fig. 5c

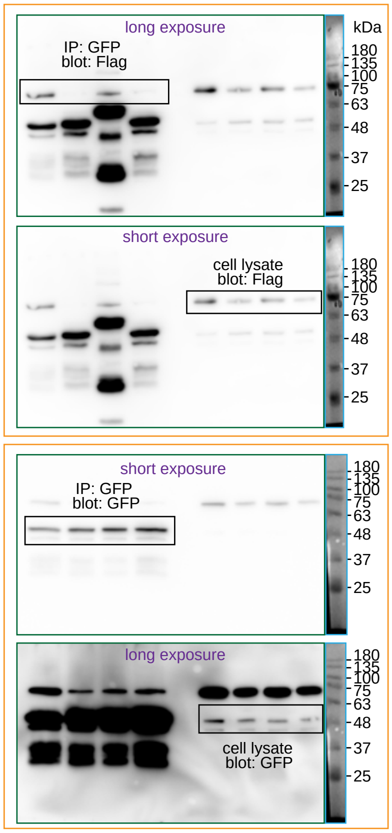

Fig. 5d

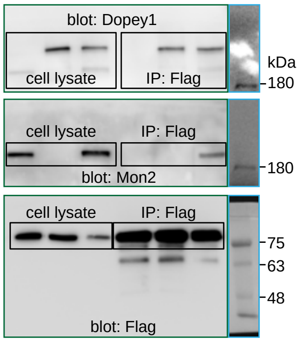

Fig. 5e

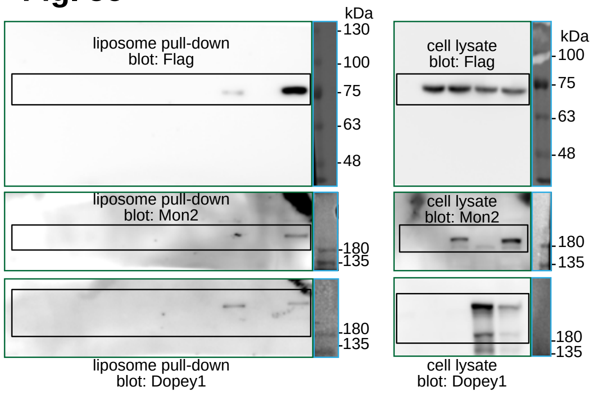

Supplementary Figure 9

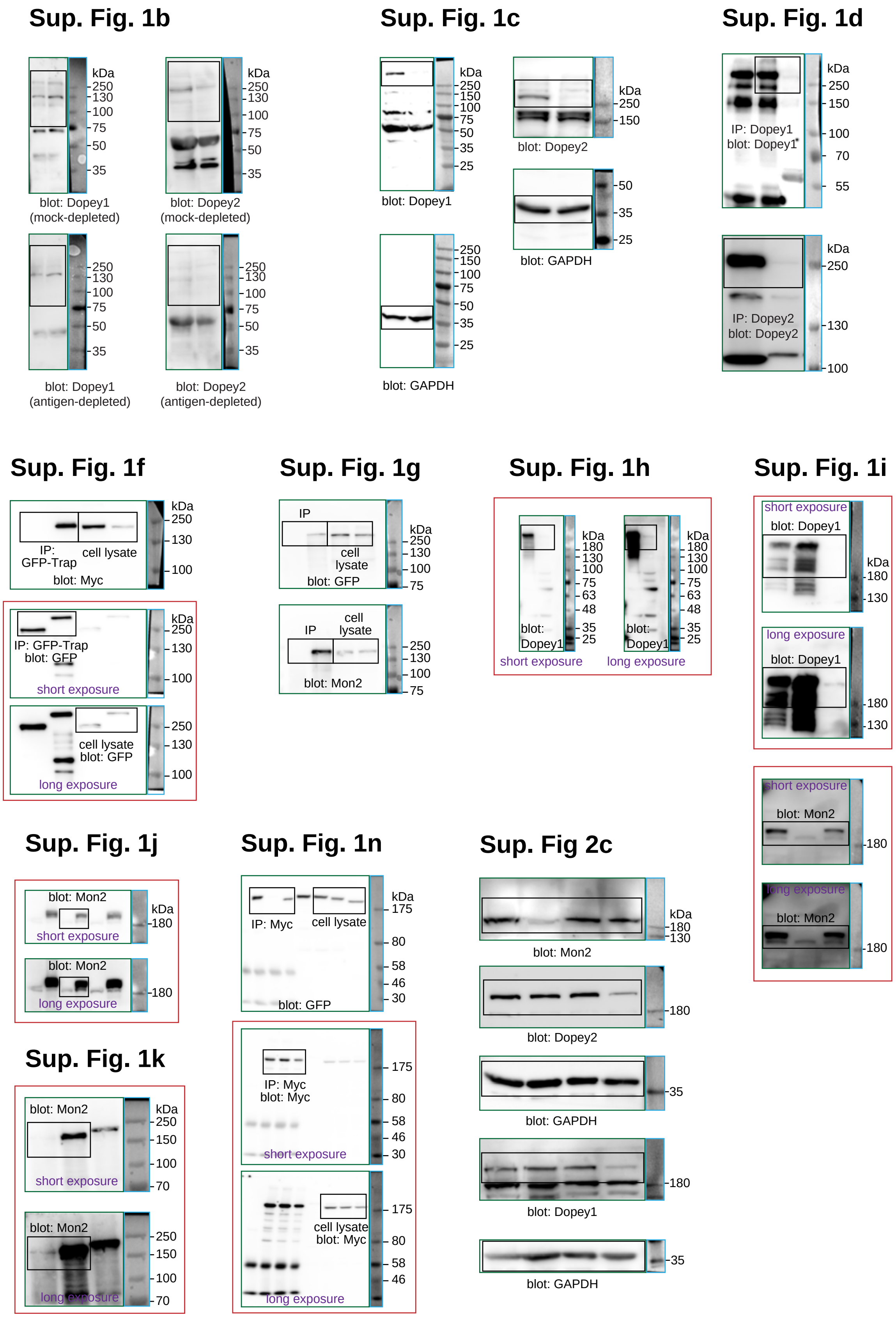

Supplementary Figure 9

Sup. Fig 2g

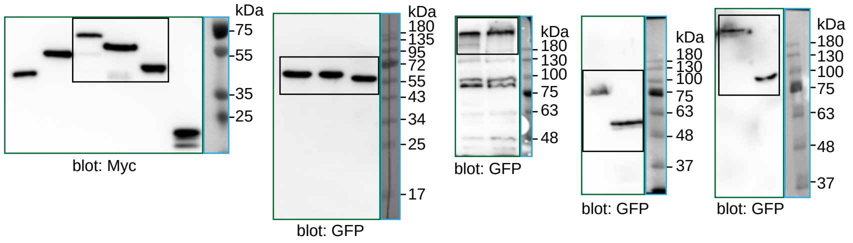

Sup. Fig 2i

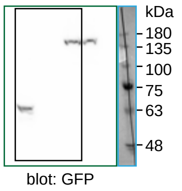

Sup. Fig. 3a

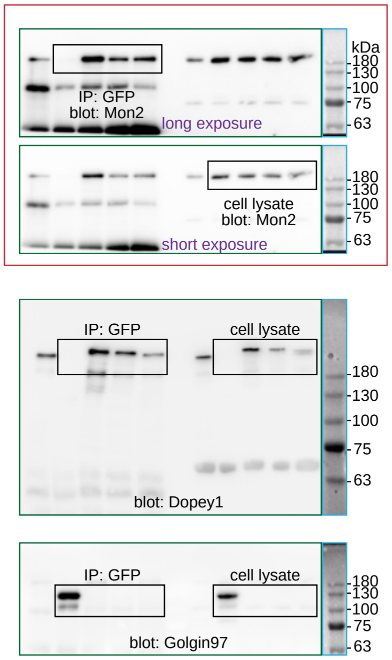

Sup. Fig. 3i

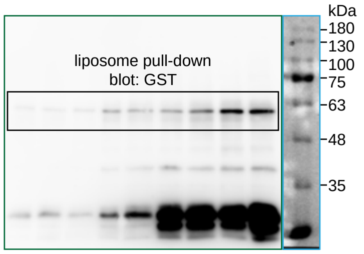

Sup. Fig. 3t

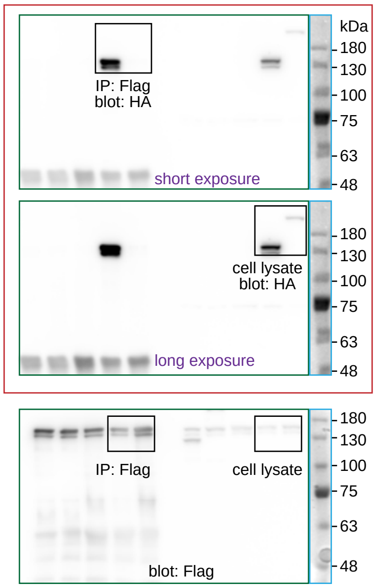

Sup. Fig. 3k

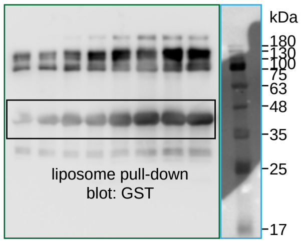

Sup. Fig. 4a

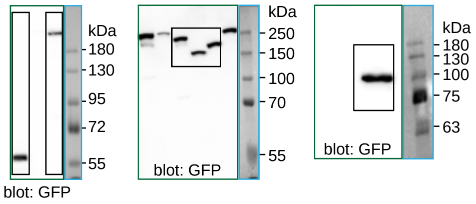

Sup. Fig. 5a

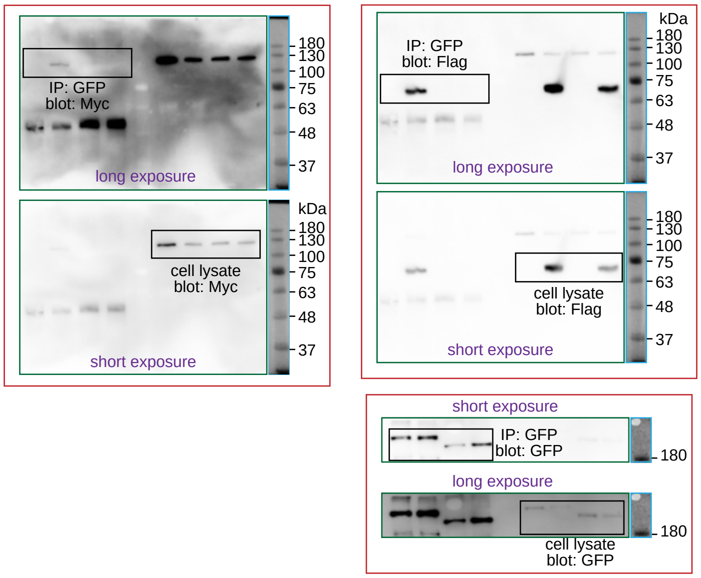

Supplementary Figure 9

Sup. Fig. 5b

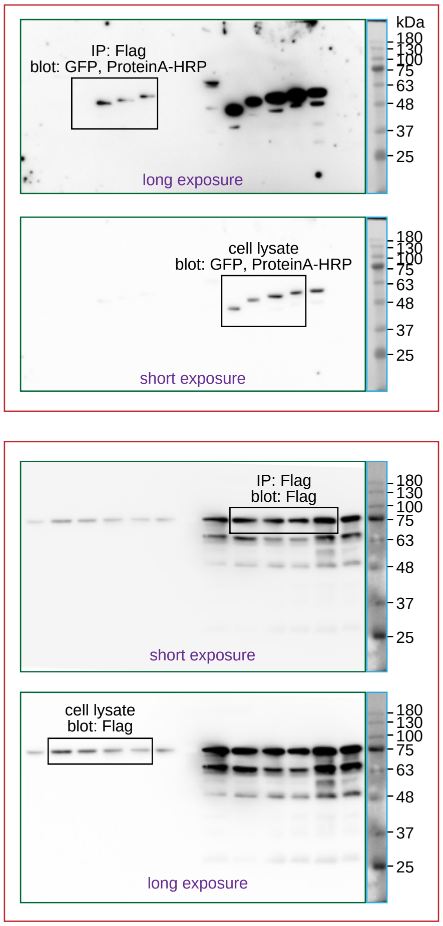

Sup. Fig. 5c

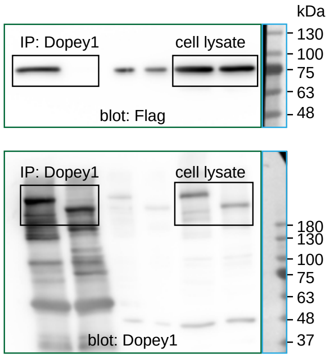

Sup. fig. 5d

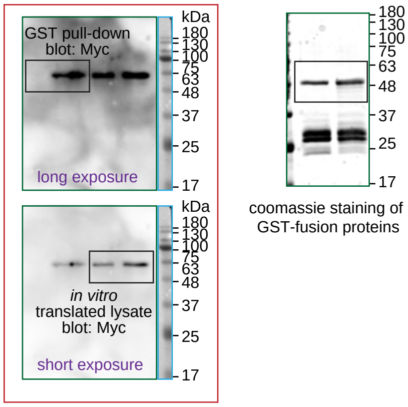

Sup. Fig. 5e

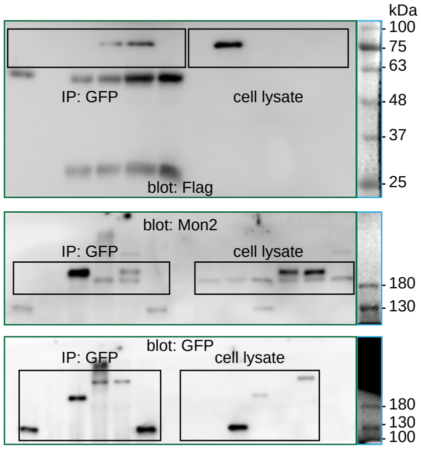

Supplementary Figure 9 Uncropped gel blot images used to prepare Fig. 1, 3, 4 and 5 and Sup. Fig. 1-5. Green box, chemiluminescence image; black box, cropped region that are shown in the corresponding figures; blue box, white light image of pre-stained molecular weight marker bands; orange box, images from the same blot but with different exposure time or blotting antibody. Molecular weight (kDa) is labeled in all blots.

Supplementary table 1: DNA plasmids

name	vector (source)	cloning sites	primers	brief method or source reference
**Vectors**				
pEGFP-C1x	pEGFP-C1x	NheI	5'-CTA GAC TTG C A-3' and 5'-CTA GTG CAA GT-3'	The two oligonucleotides were annealed and ligated into NheI digested pEGFP-C1x. NheI site was abolished in the resulting vector.
pMyc-C1x	pEGFP-C1x	AgeI/HindIII	5'-CCG GTG CCA CCA TGG AGC AGA AGC TGA TCA GCG AGG AGG ACC TGA-3' and 5'-AGC TTC AGG TCC TCC TCG CTG ATC AGC TTC TGC TCC ATG GTG GCA-3'	The two oligonucleotides were annealed and ligated into AgeI/HindIII digested pEGFP-C1x vector.
pET-30ax	pET-30a (Novagen)	BglII	5'-A GAT CCG AGA TCT GCT AGC GAA TTC CCG CGG GGA TCC T-3' and 5'-AA TTG GGA TCC CCG CGG GAA TTC GCT AGC AGA TCT CG-3'	The two oligonucleotides were annealed and ligated into BglII digested pET-30a. BglII site was abolished in the resulting vector.
**Constructs for Mon2**				
GFP-Mon2				Previous study[1]
mCherry-Mon2				Previous study[1]
DMyc-Mon2				Previous study[1]
DMyc-Mon2(1149-1432)	pDMyc-neo[1]	HindIII/EcoRI	5'-ACT GAC CTC GAG ACG CAA GCT TCG CAT ATA CAG TCA GCA GCA CTC AGC-3' and 5'-ACT GAC GCG GCC GCA CGG AAT TCT CAC ACC ACT GCT TTG TGA CAC GC -3'	The coding sequence (CDS) of a Mon2 fragment containing residues 1149-1432 was PCR amplified using GFP-Mon2 as the template. The resulting PCR product was digested by HindIII/EcoRI and ligated into pDMyc-neo vector using the same sites.
GFP-Mon2(204-1718)				Previous study[1]
GFP-Mon2(1-1634)	pEGFP-C1 (Clontech)	BamHI	5'-CAGCAAAAGCAGGCAGAAGAG-3' and 5'-CTCAGTGGATCCATTCCGCGGTTAAGGGCATTTACCACTTAATC-3'	The coding sequence (CDS) of a Mon2 fragment containing residues 1035-1634 was PCR amplified using IMAGE clone 8860514 as the template. The resulting PCR product was digested by BamHI and ligated into GFP-Mon2(1-1035) using the same sites.
GFP-Mon2(1-1200)				Previous study[1]
GFP-Mon2(1-1035)				Previous study[1]
GFP-Mon2(528-1035)	pEGFP-C3 (Clontech)	SalI/BamHI		The CDS of a Mon2 fragment containing residues 528-1035 was released by digesting GFP-Mon2 with SalI/BamHI and ligated into pEGFP-C3 vector using the same sites.

GFP-Mon2(1-528)	pEGFP-C2 (Clontech)	EcoRI/BamHI	5'-AGA CCT GAA TTC ATG TCC GGC ACC AGC AGC-3' and 5'-AGG TCA GG ATT CTG TGT TGG TGA AGA ACC-3'	The CDS of a Mon2 fragment containing residues 1-528 was PCR amplified using GFP-Mon2 as the template. The resulting PCR product was digested by EcoRI/BamHI and ligated into pEGFP-C2 vector using the same sites.
GFP-Mon2(1-228)	pEGFP-C1 (Clontech)	BglII	5'-TAT AGA TCT ATG TCC GGC ACC AGC AGC-3' and 5'-GCG AGA TCT TCT GTT ACT ATT TCC TTG-3'	The CDS of a Mon2 fragment containing residues 1-228 was PCR amplified using IMAGE clone 8860514 as the template. The resulting PCR product was digested by BglII and ligated into pEGFP-C1 vector using the same sites.
GFP-MEC	pEGFP-C3 (Clontech)	XhoI/EcoRI	5'-ACT GAC CTC GAG AGC ACA TGG AAA CTA GCA GTA-3' and 5'-ACT GAC GCG GCC GCA CGA ATT CGT CAA GAT TCT CCA TTT TGA AC-3'	The CDS of a Mon2 fragment containing residues 1458-1718 was PCR amplified using GFP-Mon2 as the template. The resulting PCR product was digested by XhoI/EcoRI and ligated into pEGFP-C3 vector using the same sites.
DMyc-MEC	pDMyc-neo[1]	XhoI/NotI	5'-ACT GAC CTC GAG AGC ACA TGG AAA CTA GCA GTA-3' and 5'-ACT GAC GCG GCC GCA CGA ATT CGT CAA GAT TCT CCA TTT TGA AC-3'	The CDS of a Mon2 fragment containing residues 1458-1718 was PCR amplified using GFP-Mon2 as the template. The resulting PCR product was digested by XhoI/NotI and ligated into pDMyc-neo vector using the same sites.
GFP-Mon2-Y4R	pEGFP-C1 (Clontech)	SalI/XbaI	5'-CA CAG TCG ACA GAA CAG CAG GAT TTA CAG TC-3', 5'-GC AAG TGG CTG GCC AAC AGC AAC AAC TTG TTG GTG AGA TTC AC-3', 5'-CAA GTT GTT GCT GTT GGC CAG CCA CTT GCA GTC CAG CCT CAA GGG ACA G-3', 5'-CAT TGC TTC TAG AGA CAA GGA GCA AAG TGC-3'	Two PCR amplifications were performed by using GFP-Mon2 as the template and the first/second and third/fourth primer pair, respectively. The two PCR fragments were mixed and subjected to the second round of PCR amplification using the first/fourth primer pair. The resulting PCR product was digested by SalI/XbaI and ligated into GFP-Mon2 using the same sites. This construct contains synonymous mutations at Mon2 siRNA #1 targeting region.
**Constructs for Dopey1**				
GFP-Dopey1	pEGFP-C1x	NotI/SacII	5'-ACT GAC GCG GCC GCT ATG AAC ACA GAA GAG CTG-3' , 5'-CAT AGA AAG CTT CCT GTT TAA ATG GGC AAG-3', 5'-TTA TCT GCT AGC ACT GGA G-3', 5'-CTG TGG AGG CTG CAC ATG CTC-3', 5'-GAG CAT GTG CAG CCT CCA CAG-3' and 5'-ATA TGG AGA TCT CTC GTT AG-3'	The CDS of Dopey1 fragment containing residues 1-210 was PCR amplified using IMAGE clone 9053072 as the template and first/second primer pair. The resulting PCR product was digested by NotI/HindIII and ligated into pEGFP-C1x vector using the same sites to obtain the intermediate clone(1-210). The CDS containing residues 210-1900 was released from IMAGE clone 9053072 using HindIII/EcoRI and ligated into clone(1-210) using the same sites to obtain the intermediate clone(1-1900). The CDS containing residues 1900-2456 was released from IMAGE clone KIAA1177 using EcoRI/SacII and ligated into clone(1-1900) using the same sites. Unfortunately, in the resulting full length Dopey1 construct, there was a nonsense C to T nucleotide mutation at residue 2328 resulting in an unnatural stop codon. The nonsense mutation was subsequently corrected by site-directed mutagenesis. Two PCR amplifications were performed by using GFP-Dopey1 as the template and the third/fourth and fifth/sixth primer pair, respectively. The two PCR fragments were mixed and subjected to the second round of PCR amplification using the first/fourth primer pair. The resulting

				PCR product was digested by NheI/EcoRI and ligated into GFP-Dopey1 using the same sites.
mCherry-Flag-Dopey1	pEGFP-C1x	AgeI/NotI	5'-ACG GAT ACC GGT GGT ACC CGC CAC CAT GGT GAG CAA GG-3' and 5'-ACG GAT GC GGC CGC CTT ATC GTC GTC ATC CTT GTA ATC CTT GTA CAG CTC GTC CAT GC-3'	The CDS of mCherry was PCR amplified using pmCherry-C1 vector as the template. The resulting PCR product was digested by AgeI/NotI and ligated into GFP-Dopey1 using the same sites. The Flag tag was introduced by the second PCR primer.
mCherry-HA-Dopey1	pEGFP-C1x	AgeI/NotI	5'-ACG GAT ACC GGT GGT ACC CGC CAC CAT GGT GAG CAA GG-3' and 5'-ACG GAT GC GGC CGC CGC GTA ATC TGG CAC ATC GTA TGG GTA CTT GTA CAG CTC GTC CAT G-3'	The CDS of mCherry was PCR amplified using pmCherry-C1 vector as the template. The resulting PCR product was digested by AgeI/NotI and ligated into GFP-Dopey1 using the same sites. The HA tag was introduced by the second PCR primer.
GFP-Dopey1(LKRL-4A)	pEGFP-C1x	EcoRI/SacII	5'-AGA TCT AGA ATT CCA GTG CCC AAT TTA GTG G-3', 5'-GC AAA TGC AGC GGC TGC GGC AAG CAT AGC TCT CTG TTC TAG-3' and 5'-GA GCT ATG CTT GCC GCA GCC GCT GCA TTT GCT ATT TTT AGC AGT G -3', 5'- ACT GAC GCGGCCGC ACG AAT TCG TCA AGT TTT TAT CAT CCC TTC-3'	Two PCR amplifications were performed by using GFP-Dopey1 as the template and the first/second and third/fourth primer pair, respectively. The two PCR fragments were mixed and subjected to the second round of PCR amplification using the first/ fourth primer pair. The resulting PCR product was digested by EcoRI/SacII and ligated into GFP-Dopey1 using the same sites. This construct contains LKRL-4A mutation.
GFP-Dopey1-R	pEGFP-C1x	HindIII/NheI	5'-G GAA GAT CAA CTT TAT ATA ATT GGT AGC GAC ATC GAG CTG ATG GTA GAA GCA GTA AGT AC-3', 5'-CAG GAT CAC TGG ACC GAG TTC AGG TTT GTC CAG TAA ACT GAT TAA AAT GCG-3', 5'-G GAC AAA CCT GAA CTC GGT CCA GTG ATC CTG GAA GAT GTC CTG ATT GAA G-3', 5'-TCC AGT GCT AGC AGA TAA CAG TGG AGG-3', 5'- AAC AGG AAG CTT TCT ATG GAA GAT CAA CTT TAT ATA ATT GGT AGC GAC ATC GAG CTG ATG-3',	Two PCR amplifications were performed by using GFP-Dopey1 as the template and the first/second and third/fourth primer pair, respectively. The two PCR fragments were mixed and subjected to the second round of PCR amplification using the fifth/ fourth primer pair. The resulting PCR product was digested by HindIII/NheI and ligated into GFP-Dopey1(1-1900) using the same sites. The CDS for residues 1900-2456 was released from GFP-Dopey1(1900-2456) using EcoRI/SacII and ligated into GFP-Dopey1(1-1900) generated above using the same sites. This construct contains synonymous mutations at Dopey1 siRNA #1 and #2 targeting region.
Myc-Dopey1(540-1900)	pMyc-C1x	NheI/EcoRI	5'-AG CTT ACT AGT AGA GAT CTC G -3' and 5'-CT AGC GAG ATC TCT ACT AGT A -3'	The CDS of a Dopey1 fragment containing residues 210-1900 was released by digesting GFP-Dopey1 with HindIII/EcoRI and ligated into pMyc-C1x vector using the same sites to obtain Myc-Dopey1(210-1900). The two oligonucleotides were annealed and ligated into HindIII/NheI digested Myc-Dopey1(210-1900).
GFP-DEC	pEGFP-C3 (Clontech)	EcoRI/BamHI	5'- ACT GAC AGA ATT CGT GTG GCT CAA AGC AGT TCA C-3', 5'- A CTG AC GGA TCC TCA AGT TTT TAT CAT CCC TTC CAG-3'	The CDS of a Dopey1 fragment containing residues 2146-2456 was PCR amplified using GFP-Dopey1 as the template. The resulting PCR product was digested by EcoRI/BamHI and ligated into pEGFP-C3 vector using the same sites.

GFP-Dopey1(1-100)	pEGFP-C2 (Clontech)	EcoRI/BamHI	5'- ACG GAC GAA TTC ATG AAC ACA GAA GAG CTG GAG-3' and 5'-ACG GAT GGA TCC CCG CGG GTC GAC TTA GGT ACC GGC AAG TCG CTT AGG TCC-3'	The CDS of a Dopey1 fragment containing residues 1-100 was PCR amplified using GFP-Dopey1 as the template. The resulting PCR product was digested by EcoRI/BamHI and ligated into pEGFP-C2 vector using the same sites.
GFP-Dopey1(1-148)	pEGFP-C2 (Clontech)	EcoRI/BamHI	5'- ACG GAC GAA TTC ATG AAC ACA GAA GAG CTG GAG-3' and 5'-ACG GAT GGA TCC CCG CGG GTC GAC TTA GGT ACC AAG CAA TCC CTG TAG ACC AGG-3'	The CDS of a Dopey1 fragment containing residues 1-148 was PCR amplified using GFP-Dopey1 as the template. The resulting PCR product was digested by EcoRI/BamHI and ligated into pEGFP-C2 vector using the same sites.
GFP-Dopey1(1-175)	pEGFP-C2 (Clontech)	EcoRI/BamHI	5'- ACG GAC GAA TTC ATG AAC ACA GAA GAG CTG GAG-3' and 5'-ACG GAT GGA TCC CCG CGG GTC GAC TTA GGT ACC CAC AGC AGC AGC AAC CTT TTC C-3'	The CDS of a Dopey1 fragment containing residues 1-175 was PCR amplified using GFP-Dopey1 as the template. The resulting PCR product was digested by EcoRI/BamHI and ligated into pEGFP-C2 vector using the same sites.
GFP-DEN	pEGFP-C2 (Clontech)	EcoRI/BamHI	5'- ACG GAC GAA TTC ATG AAC ACA GAA GAG CTG GAG-3' and 5'-ACG GAT GGA TCC CCG CGG GTC GAC TTA GGT ACC AAG CTT CCT GTT TAA ATG GGC-3'	The CDS of a Dopey1 fragment containing residues 1-210 was PCR amplified using GFP-Dopey1 as the template. The resulting PCR product was digested by EcoRI/BamHI and ligated into pEGFP-C2 vector using the same sites.
GFP-DEN(W34A)	pEGFP-C2 (Clontech)	EcoRI/BamHI	5'-ACG GAC GAA TTC ATG AAC ACA GAA GAG CTG GAG-3', 5'-CGC TTC ACT GGA GTA TTC-3', 5'-GAA TAC TCC AGT GAA GCG GCA GAT TTG ATA TCA GCA C-3', 5'-ACG GAT GGA TCC CCG CGG GTC GAC TTA GGT ACC AAG CTT CCT GTT TAA ATG GGC-3'	Two PCR amplifications were performed by using GFP-DEN as the template and the first/second and third/fourth primer pair, respectively. The two PCR fragments were mixed and subjected to the second round of PCR amplification using the first/fourth primer pair. The resulting PCR product was digested by EcoRI/BamHI and ligated into pEGFP-C2 using the same sites. This construct contains W34A mutation.
GFP-DEN(W186A)	pEGFP-C2 (Clontech)	EcoRI/BamHI	5'-ACG GAC GAA TTC ATG AAC ACA GAA GAG CTG GAG-3', 5'-CT GGT GAG AAG ACT ACC TGC CAG GGC ACT GTA GAA TGC-3', 5'-GCA GGT AGT CTT CTC ACC AG-3', 5'-ACG GAT GGA TCC CCG CGG GTC GAC TTA GGT ACC AAG CTT CCT GTT TAA ATG GGC-3'	Two PCR amplifications were performed by using GFP-DEN as the template and the first/second and third/fourth primer pair, respectively. The two PCR fragments were mixed and subjected to the second round of PCR amplification using the first/fourth primer pair. The resulting PCR product was digested by EcoRI/BamHI and ligated into pEGFP-C2 vector using the same sites. This construct contains W186A mutation.
GFP-DEN(WW-AA)	pEGFP-C2 (Clontech)	EcoRI/BamHI	5'-ACG GAC GAA TTC ATG AAC ACA GAA GAG CTG GAG-3', 5'-CGC TTC ACT GGA GTA TTC-3', 5'-GAA TAC TCC AGT GAA GCG GCA GAT TTG ATA TCA GCA C-3', 5'-CT GGT GAG AAG ACT ACC TGC CAG GGC ACT GTA GAA TGC-3', 5'-GCA GGT AGT CTT CTC ACC AG-3', 5'-GCC GAT TTC GGC CTA TTG G-3', 5'-ACG GAT GGA TCC CCG CGG GTC GAC TTA GGT ACC AAG CTT CCT GTT TAA ATG GGC-3'	Three PCR amplifications were performed by using GFP-DEN as the template and the first/second, third/fourth and fifth/sixth primer pair, respectively. The three PCR fragments were mixed and subjected to the second round of PCR amplification using the first/seventh primer pair. The resulting PCR product was digested by EcoRI/BamHI and ligated into pEGFP-C2 vector using the same sites. This construct contains WW-AA mutation.
GFP-Dopey1(1-1900)	pEGFP-C1x	EcoRI/SacII	5'-AA TTC GGT ACC AGT AAC TCG AGG TCG ACC CGC-3' and 5'- GGG TCG ACC TCG AGT TAC TGG TAC CG-3'	The two oligonucleotides were annealed and ligated into EcoRI/SacII digested GFP-Dopey1.
GFP-Dopey1(210-1900)	pEGFP-C1x	AgeI/HindIII	5'-ACG AGT ACC GGT GCC ACC ATG GTG AGC AAG GGC GAG GAG-3' and 5'- ACG AGT AAG CTT GGC ATG GAC GAG CTG TAC AAG-3'	The CDS of GFP was PCR amplified using pEGFP-C1x vector as the template. The resulting PCR product was digested by AgeI/HindII and ligated into GFP-Dopey1(1-1900) using the same sites.

GFP-Dopey1(1-776)	pEGFP-C1x			GFP-Dopey1 with the nonsense mutation at residue 777 (CAG to TAG). It was serendipitously obtained during our cloning of GFP-Dopey1.
DMyc-Dopey1(894-1247)	pDMyc-neo[1]	EcoRI/SalI	5'-A CTG ACG AAT TC A GTT CCT TCT TCT AGC ATC-3' and 5'-A CTG ACG TCG ACT GGT TTC TAT GGA AGC AAC-3'	The CDS of a Dopey1 fragment containing residues 894-1247 was PCR amplified using GFP-Dopey1 as the template. The resulting PCR product was digested by EcoRI/SalI and ligated into pDMyc-neo vector using the same sites.
DMyc-Dopey1(1240-1586)	pDMyc-neo[1]	EcoRI/SalI	5'-A CTG ACG AAT TC T GAC TCT TCT GTT GCT TCC-3' and 5'-A CTG ACG TCG ACT CTG TGT TCT AGA ACA ATC-3'	The CDS of a Dopey1 fragment containing residues 1240-1586 was PCR amplified using GFP-Dopey1 as the template. The resulting PCR product was digested by EcoRI/SalI and ligated into pDMyc-neo vector using the same sites.
DMyc-Dopey1(1570-1894)	pDMyc-neo[1]	EcoRI/SalI	5'-A CTG ACG AAT TC G TCA CAA CTT CTT AAG GTG-3' and 5'-A CTG ACG TCG ACA GCA TAG AAA AAC TGA AGC-3'	The CDS of a Dopey1 fragment containing residues 1570-1894 was PCR amplified using GFP-Dopey1 as the template. The resulting PCR product was digested by EcoRI/SalI and ligated into pDMyc-neo vector using the same sites.
GFP-Dopey1(1900-2456)	pEGFP-C3 (Clontech)	EcoRI/SacII	5'-AGA TCT AGA ATT CCA GTG CCC AAT TTA GTG G-3' and 5'-ACT GAC GCGGCCGC ACG AAT TCG TCA AGT TTT TAT CAT CCC TTC-3'	The CDS of a Dopey1 fragment containing residues 1900-2456 was PCR amplified using GFP-Dopey1 as the template. The resulting PCR product was digested by EcoRI/SacII and ligated into pEGFP-C3 vector using the same sites.
GFP-Dopey1(1900-2456)(LKRL-4A)	pEGFP-C3 (Clontech)	EcoRI/SacII	5'-AGA TCT AGA ATT CCA GTG CCC AAT TTA GTG G-3' 5'-GA GCT ATG CTT GCC GCA GCC GCT GCA TTT GCT ATT TTT AGC AGT G-3', 5'-GC AAA TGC AGC GGC TGC GGC AAG CAT AGC TCT CTG TTC TAG-3' and 5'-ACT GAC GCGGCCGC ACG AAT TCG TCA AGT TTT TAT CAT CCC TTC-3'	Two PCR amplifications were performed by using GFP-Dopey1 as the template and the first/second and third/fourthprimer pair, respectively. The two PCR fragments were mixed and subjected to the second round of PCR amplification using the first/fourth primer pair. The resulting PCR product was digested by EcoRI/SacII and ligated into pEGFP-C3 vector using the same sites. This construct contains LKRL-4A mutation.
GFP-Dopey1(2101-2408)	pEGFP-C3 (Clontech)	XhoI/EcoRI	5'- ACT GAC CTC GAG GAA GCT TTT GAC CTC TTT ATG-3' and 5'-ACT GAC GCGGCCGC ACG AAT TCG TCA TCC TCC ACA TCG GCT TGT GAC-3'	The CDS of a Dopey1 fragment containing residues 2101-2408 was PCR amplified using GFP-Dopey1 as the template. The resulting PCR product was digested by XhoI/EcoRI and ligated into pEGFP-C3 vector using the same sites.
GFP-Dopey1(2126-2456)	pEGFP-C3 (Clontech)	XhoI/EcoRI	5'-ACT GAC CTC GAG ATG GAC AAT CTG ATG ACA-3' and 5'-ACT GAC GCGGCCGC ACG AAT TCG TCA AGT TTT TAT CAT CCC TTC-3'	The CDS of a Dopey1 fragment containing residues 2126-2456 was PCR amplified using GFP-Dopey1 as the template. The resulting PCR product was digested by XhoI/EcoRI and ligated into pEGFP-C3 vector using the same sites.
GFP-Dopey1(2182-2456)	pEGFP-C3 (Clontech)	XhoI/EcoRI	5'-ACT GAC CTC GAG GAC CAG TAC CAG AAA TAT C-3' and 5'-ACT GAC GCGGCCGC ACG AAT TCG TCA AGT TTT TAT CAT CCC TTC-3'	The CDS of a Dopey1 fragment containing residues 2182-2456 was PCR amplified using GFP-Dopey1 as the template. The resulting PCR product was digested by XhoI/EcoRI and ligated into pEGFP-C3 vector using the same sites.

GFP-DEC(2146-2456)(LKRL-4A)	pEGFP-C3 (Clontech)	XhoI/EcoRI	5'-ACT GAC CTC GAG ATG GAC AAT CTG ATG ACA-3', 5'-GC AAA TGC AGC GGC TGC GGC AAG CAT AGC TCT CTG TTC TAG-3' and 5'-GA GCT ATG CTT GCC GCA GCC GCT GCA TTT GCT ATT TTT AGC AGT G -3', 5'- ACT GAC GCGGCCGC ACG AAT TCG TCA AGT TTT TAT CAT CCC TTC-3'	Two PCR amplifications were performed by using GFP-Dopey1 as the template and the first/second and third/fourthprimer pair, respectively. The two PCR fragments were mixed and subjected to the second round of PCR amplification using the first/fourth primer pair. The resulting PCR product was digested by XhoI/EcoRI and ligated into pEGFP-C3 vector using the same sites. This construct contains LKRL-4A mutation.
GFP-tdDEC	pEGFP-C3 (Clontech)	XhoI/EcoRI	5'- ACT GAC AGA ATT CGT GTG GCT CAA AGC AGT TCA C-3', 5'- A CTG AC GGA TCC TCA AGT TTT TAT CAT CCC TTC CAG-3', 5'-ACT GAC CTC GAG GTG GCT CAA AGC AGT TCA C-3', 5'- A CTG ACG AAT TCG AGT TTT TAT CAT CCC TTC CAG-3'	The CDS of a Dopey1 fragment comprising residues 2146-2456 was PCR amplified twice using GFP-Dopey1 as the template and the first/second and third/fourth primer pairs. The resulting two PCR products were digested by EcoRI/BamHI and XhoI/EcoRI, respectively, and sequentially ligated into pEGFP-C2 vector using the same sites.
GFP-Dopey1ΔDEC	pEGFP-C3 (Clontech)	EcoRI/SacII	5'-AG ATC TGA ATT CCA GTG CCC AAT TTA GTG G-3' and 5'-AGA TCT CCG CGG CTC GAG TTA GGT ACC TGC TAC ACG AGT CAT CAA ATC TC-3'	The CDS of a Dopey1 fragment containing residues 1900-2146 was PCR amplified using GFP-Dopey1 as the template. The resulting PCR product was digested by EcoRI/SacII and ligated into GFP-Dopey1 using the same sites.
GST-DEC	pGEB[1]	EcoRI/BamHI	5'-AGC TCT GAA TTC GAG CAG AAG CTG ATC AGC GAG GAG GAC CTG ATG GAC AAT CTG ATG AC-3' and 5'-AGG TCG TCT AGA GGA TCC CCG CGG TCA AGT TTT TAT CAT CCC TTC-3'	The CDS of a Dopey1 fragment containing residues 2126-2456 was PCR amplified using GFP-Dopey1 as the template. The resulting PCR product was digested by EcoRI/BamHI and ligated into pGEB vector using the same sites. The Myc tag was introduced by the second PCR primer.
GST-DEC(LKRL-4A)	pGEB[1]	EcoRI/BamHI	5'-AGC TCT GAA TTC GAG CAG AAG CTG ATC AGC GAG GAG GAC CTG ATG GAC AAT CTG ATG AC-3' and 5'-AGG TCG TCT AGA GGA TCC CCG CGG TCA AGT TTT TAT CAT CCC TTC-3'	The CDS of a Dopey1 fragment containing residues 2126-2456 was PCR amplified using GFP-Dopey1 as the template. The resulting PCR product was digested by EcoRI/BamHI and ligated into pGEB vector using the same sites. This construct contains LKRL-4A mutation. The Myc tag was introduced by the second PCR primer.
mCherry-Flag-Dopey1-ACC1	pEGFPC1-x	XbaI/EcoRI	5'-A CGG ATT CTA GAA GGA TCC GCC ACC ATG GAA GGT G-3' and 5'-A CGG ATG AAT TCT CAT GGT GGC GAC CGG CGA G-3'	The CDS of ACC1 was PCR amplified using Spo20-ACC1-mCherry (a gift from Bruno Antony[2]) as the template. The resulting PCR product was digested by XbaI/EcoRI and ligated into mCherry-Flag-Dopey1 using the same sites.
mCherry-HA-Dopey1-ACC1	pEGFPC1-x	XbaI/EcoRI	5'-A CGG ATT CTA GAA GGA TCC GCC ACC ATG GAA GGT G-3' and 5'-A CGG ATG AAT TCT CAT GGT GGC GAC CGG CGA G-3'	The CDS of ACC1 was PCR amplified using Spo20-ACC1-mCherry as the template. The resulting PCR product was digested by XbaI/EcoRI and ligated into mCherry-HA-Dopey1 using the same sites.
GFP-Dopey1ΔDEC-FRB	pEGFP-C3 (Clontech)	KpnI/SacII	5'-AGA TCT GGT ACC ATC CTC TGG CAT GAG ATG TGG-3' and<br>5'-AGA TCT CCG CGG TTA CTT TGA GAT TCG TCG GAA CAC-3'	The CDS of FRB was PCR amplified using iRFP-FRB-Rab6 (Addgene #51612; a gift from Tamas Balla[3]) as the template. The resulting PCR product was digested by KpnI/SacII and ligated into GFP-Dopey1ΔDEC using the same sites.
GFP-FRB	pEGFP-C3 (Clontech)	KpnI/SacII	5'-AGA TCT GGT ACC ATC CTC TGG CAT GAG ATG TGG-3' and<br>5'-AGA TCT CCG CGG TTA CTT TGA GAT TCG TCG GAA CAC-3'	The CDS of FRB was PCR amplified using iRFP-FRB-Rab6 as the template. The resulting PCR product was digested by KpnI/SacII and ligated into pEGFP-C3 vector using the same sites.

GFP-Dopey1(1900-2146)-FRB	pEGFP-C3 (Clontech)	EcoRI/SacII	5'- AG ATC TGA ATT CCA GTG CCC AAT TTA GTG G-3' and 5' AGA TCT CCG CGG CTC GAG TTA GGT ACC TGC TAC-3'	The CDS of a Dopey1 fragment containing residues 1900-2146 was PCR amplified using GFP-Dopey1 as the template. The resulting PCR product was digested by EcoRI/SacII and ligated into GFP-FRB using the same sites.
GFP-DEN-FRB	pEGFP-C2 (Clontech)	KpnI/SacII	5'-AGA TCT GGT ACC ATC CTC TGG CAT GAG ATG TGG-3' and 5'-AGA TCT CCG CGG TTA CTT TGA GAT TCG TCG GAA CAC-3'	The CDS of FRB was PCR amplified using iRFP-FRB-Rab6 as the template. The resulting PCR product was digested by KpnI/SacII and ligated into GFP-DEN using the same sites.
GFP-DEN(W34A)-FRB	pEGFP-C2 (Clontech)	KpnI/SacII	5'-AGA TCT GGT ACC ATC CTC TGG CAT GAG ATG TGG-3' and 5'-AGA TCT CCG CGG TTA CTT TGA GAT TCG TCG GAA CAC-3'	The CDS of FRB was PCR amplified using iRFP-FRB-Rab6 as the template. The resulting PCR product was digested by KpnI/SacII and ligated into GFP-DEN(W34A) using the same sites. This construct contains W34A mutation.
GST-DEN	pGEB[1]	EcoRI/BamHI	5'-ACG GAC GAA TTC ATG AAC ACA GAA GAG CTG GAG-3', 5'-ACG GAT GGA TCC CCG CGG GTC GAC TTA GGT ACC AAG CTT CCT GTT TAA ATG GGC-3'	The CDS of a Dopey1 fragment containing residues 1-210 was PCR amplified using GFP-Dopey1 as the template. The resulting PCR product was digested by EcoRI/BamHI and ligated into pGEB vector using the same sites.
GST-DEN(W34A)	pGEB[1]	EcoRI/BamHI	5'-ACG GAC GAA TTC ATG AAC ACA GAA GAG CTG GAG-3', 5'-ACG GAT GGA TCC CCG CGG GTC GAC TTA GGT ACC AAG CTT CCT GTT TAA ATG GGC-3'	The CDS of a Dopey1 fragment containing residues 1-210 was PCR amplified using GFP-DEN(W34A) as the template. The resulting PCR product was digested by EcoRI/BamHI and ligated into pGEB vector using the same sites.
Dopey1(1171-1320)-6×His	pET30a (Novagen)	NdeI/XhoI	5'-ACT GAC CAT ATG CCC CCA AAG TGC AGT GAT ATA G-3' and 5'-GGA GAC CTC GAG CAG ACC ATC ACT GAA GAA TAC-3'	The CDS of a Dopey1 fragment containing residues 1171-1320 was PCR amplified using GFP-Dopey1 as the template. The resulting PCR product was digested by NdeI/XhoI and ligated into pET30a vector using the same sites.
GST-Dopey1(1171-1320)	pGEB[1]	EcoRI/BamHI	5'-ACT GAC CAT ATG CCC CCA AAG TGC AGT GAT ATA G-3' and 5'-GGA GAC CTC GAG CAG ACC ATC ACT GAA GAA TAC-3'	The CDS of a Dopey1 fragment containing residues 1171-1320 was PCR amplified using GFP-Dopey1 as the template. The resulting PCR product was digested by EcoRI/BamHI and ligated into pGEB vector using the same sites.
mCherry-HA-Dopey1-ACC1(W34A)	pEGFPC1-x	NotI/XbaI	5'-ACT GAC GCG GCC GCT ATG AAC ACA GAA GAG CTG-3' and 5'-ACG GA TTC TAG AGG CCT GTT TAA ATG GGC AAG AAC ATA CG-3'	The CDS of a Dopey1 fragment containing residues 1-363 was PCR amplified using GST-DEN(W34A) as the template. The resulting PCR product was digested by NotI/XbaI and ligated into mCherry-HA-Dopey1-ACC1 using the same sites. This construct contains W34A mutation.
mCherry-HA-Dopey1-ACC1(LKRL-4A)	pEGFPC1-x	EcoRI/SacII		The CDS of a Dopey1 fragment containing residues 1900-2456 was released by digesting GFP-Dopey1(1900-2456)(LKRL-A4) with EcoRI/SacII and ligated into mCherry-HA-Dopey1-ACC1 using the same sites. This construct contains LKRL-4A mutation.

PEX3-mRFP-FKBP	pCI-neo (Promega)	EcoRI/XbaI	5’-CTG GGG AAA TAT GGA CAG AAG AAA ATC AGA GAA ATA ACC GGT-3', 5’-AGA TCT TCT AGA TTA TTC CAG TTT TAG AAG CTC CAC-3', 5’-AGA TCT GAA TTC ATG CTG AGG TCT GTA TGG AAT TTT CTG AAA CGC CAC-3', 5’-GG AAT TTT CTG AAA CGC CAC AAA AAG AAA TGC ATC TTC CTG GGC ACG GTC CTT GGA GG-3' and 5’-GGC ACG GTC CTT GGA GGA GTA TAT ATT CTG GGG AAA TAT GGA CAG AAG AAA ATC AGA G-3'	PCR amplifications was performed by using Pseudojanin plasmid (Addgene #37999; a gift from Robert Irvine[4]) as the template and first/second primer pair. The PCR fragment was mixed and subjected to the second round of PCR amplification using the second/third/fourth and fifth primer. The resulting PCR product was digested by EcoRI/XbaI and ligated into pCI-neo vector using the same sites.
**Constructs for Dopey2**				
Dopey2-Myc-Flag				Purchased from Origene (Cat. no. RC218627).
GFP-Dopey2(2010-2298)	pEGFP-C3 (Clontech)	XhoI/SalI	5’-ACG GAC CTC GAG ATG AAC ATG CAG AGC AGT TC-3’ and 5’-ACG GAC GCGGCCGC ACG TCG ACG TCA ACA TTC TGG ATG TTC CAG-3’	The CDS of a Dopey2 fragment containing residues 2010-2298 was PCR amplified using Dopey2-Myc-flag as the template. The resulting PCR fragment was digested by XhoI/SalI and ligated into pEGFP-C3 vector using the same sites.
GFP-Dopey2(1169-2298)	pEGFP-C2 (Clontech)	HindIII/SacII		Dopey2-Myc-Flag was digested with HindIII/SacII and the insert was ligated into pEGFP-C2 vector using the same sites.
Dopey2(1005-1188)-6×His	pET30a (Novagen)	NdeI/XhoI	5'-ACG CTC CAT ATG GAA TTC CAG CCA AAA ACC CAG AGA ACC-3' and 5'-GAT GCA CTC GAG GGA TCC AGC CTG CGT CTT GTC CGA G-3'	The CDS of a Dopey2 fragment containing residues 1005-1188 was PCR amplified using Dopey2-Myc-Flag as the template. The resulting PCR product was digested by NdeI/XhoI and ligated into pET30a vector using the same sites.
GST-Dopey2(1005-1188)	pGEB[1]	EcoRI/BamHI	5'-ACG CTC CAT ATG GAA TTC CAG CCA AAA ACC CAG AGA ACC-3' and 5'-GAT GCA CTC GAG GGA TCC AGC CTG CGT CTT GTC CGA G-3'	The CDS of a Dopey2 fragment containing residues 1005-1188 was PCR amplified using Dopey2-Myc-Flag as the template. The resulting PCR product was digested by EcoRI/BamHI and ligated into pGEB vector using the same sites.
**Constructs for kinesin-1**				
Flag-KLC2				Purchased from GenScript (Cat. no. OHu07192).
GFP-Kif5b	pEGFP-C1x	NotI/KpnI	5'-AGG TCC GCG GCC GCT GAT TAC AAG GAT GAC GAC GAT AAG G-3' and 5'-CTG CAC GGT ACC CAC TTG TTT GCC TCC TCC AC-3'	The CDS of Kif5b was PCR amplified using Flag-Kif5b (Purchased from GenScript Cat. no. OHu14181) as the template. The resulting PCR product was digested by NotI/KpnI and ligated into GFP-Dopey1ΔDEC-FRB using the same sites.
DMyc-Kif5b	pDMyc-neo[1]	XhoI/XbaI	5’-AGG TCC CTC GAG TAC CCA TAC GAT GTT CCA GAT TAC GCT GCG GAC CTG GCC GAG TGC-3’ and 5’-CTG CAC TCT AGA CTA CAC TTG TTT GCC TCC TCC AC-3'	The CDS of Kif5b was PCR amplified using Flag-Kif5b as the template. The resulting PCR product was digested by XhoI/XbaI and ligated into pDMyc-neo vector using the same sites. The HA tag was introduced by the first PCR primer.
DMyc-TPR	pDMyc-neo[1]	EcoRI/SalI	5’-A GAC CAG AAT TCG AAG GGG GAC GTC CCC AAA GAC-3' and 5’-AGA CTC GTC GAC TTA GCC CAC CAG GGA GCT TCG-3'	The CDS of a KLC2 fragment containing its TPR domain (residues 155-622) was PCR amplified using Flag-KLC2 as the template. The resulting PCR product was digested by

				EcoRI/SalI and ligated into pDMyc-neo vector using the same sites.
**Constructs for knockdown**				
control shRNA	pLKO.1 (Addgene #10878)	AgeI/EcoRI	5'-CCG GAA CGT ACG CGG AAT ACT TCG ACT CGA GTC GAA GTA TTC CGC GTA CGT TTT TTT G-3' and 5'-AAT TCA AAA AAA CGT ACG CGG AAT ACT TCG ACT CGA GTC GAA GTA TTC CGC GTA CGT T-3'	The two oligonucleotides were annealed and ligated into AgeI/EcoRI digested pLKO.1. The shRNA has the same targeting sequence as GL2 control siRNA.
PI4KIIIβ shRNA#1	pLKO.1 (Addgene #10878)	AgeI/EcoRI	5'-CCG GAA CTC CAA GGA GCC TGG AGT ACT CGA GTA CTC CAG GCT CCT TGG AGT TTT TTT G-3' and 5'-AAT TCA AAA AAA CTC CAA GGA GCC TGG AGT ACT CGA GTA CTC CAG GCT CCT TGG AGT T-3'	The two oligonucleotides were annealed and ligated into AgeI/EcoRI digested pLKO.1.
PI4KIIIβ shRNA#2	pLKO.1 (Addgene #10878)	AgeI/EcoRI	5'-CCG GAA GCT ACG GAA GCT GAT CCT CCT CGA GGA GGA TCA GCT TCC GTA GCT TTT TTT G-3' and 5'-AAT TCA AAA AAA GCT ACG GAA GCT GAT CCT CCT CGA GGA GGA TCA GCT TCC GTA GCT T-3'	The two oligonucleotides were annealed and ligated into AgeI/EcoRI digested pLKO.1.
**Other constructs**				
GFP-P4M	pEGFP-C2 (Clontech)	XhoI/EcoRI	5'-AT GGT AGT CGA CAC TCG AGC GAA TTC CTC AGA TCC ACG GCA AGC ACG G-3' and 5'-AT GGT GGA TCC TCA GAA TTC TTT TAT CTT AAT GGT TTG TC-3'	The CDS of P4M was PCR amplified using mCherry-P4M (Addgene #51471; a gift from Tamas Balla[3]) as the template. The resulting PCR product was digested by XhoI/EcoRI and ligated into pEGFP-C2 vector using the same sites.
GST-P4M	pGEB[1]	EcoRI/BamHI	5'-AT GGT GAA TTC GAG CAG AAG CTG ATC AGC GAG GAG GAC CTG CTC AGA TCC ACG GCA AGC ACG G-3' and 5'-AT GGT GGA TCC TCA TTT TAT CTT AAT GGT TTG TC-3'	The CDS of P4M was PCR amplified using mCherry-P4M as the template. The resulting PCR product was digested by EcoRI/BamHI and ligated into pGEB vector using the same sites. The Myc tag was introduced by the first PCR primer.
mCherry-Rab7	pmCherry-C3 (Clontech)	NheI/HindIII		The CDS of mCherry was relased by digesting mCherry-Rab5 (a gift from T. Kirchhausen) using NheI/HindIII and ligated into GFP-Rab7 (a gift from T. Kirchhausen) using the same sites.
Lamp1-mCherry	pmCherry-N1 (Clontech)	BamHI/NotI		The CDS of mCherry was released by digesting pmCherry-N1 vector using BamHI/NotI and ligated into Lamp1-GFP (a gift from T. Kirchhausen) using the same sites.
GST-GFP	pGEB[1]	EcoRI/BamHI	5'-AGT GAC GAA TTC ATG GTG AGC AAG GGC GAG GAG CTG-3' and 5'-AGT GAC GGA TCC TTA CTT GTA CAG CTC GTC CAT GCC-3'	The CDS of GFP was PCR amplified using pEGFP-C2 vector as the template. The resulting PCR product was digested by EcoRI/BamHI and ligated into pGEB vector using the same sites.
GST-His	pET30ax	EcoRI/BamHI	5'-AGT GAC GAA TTC ATG GTG AGC AAG GGC GAG GAG CTG-3' and 5'-AGT GAC GGA TCC TTA CTT GTA CAG CTC GTC CAT GCC-3'	The CDS of GFP was PCR amplified using pEGFP-C2 vector as the template. The resulting PCR product was digested by

				EcoRI/BamHI and ligated into pET30ax vector using the same sites.
GFP-Rab6	pEGFP-C2 (Clontech)	EcoRI/BamHI	5'-GTA CCT GAA TTC ATG TCC ACG GGC GGA GAC TTC G-3', 5'-CAA GTC GGA TCC TTA GCA GGA ACA GCC TCC TTC AC-3'	The CDS of Rab6 was PCR amplified using IMAGE clone 2124966 as the template. The resulting PCR product was digested by EcoRI/BamHI and ligated into pEGFP-C2 vector using the same sites.
GFP-Golgin97				A gift from W. Hong[5]
VSVGtso45-GFP				A gift from J. Lippincott-Schwartz[6]
GFP-ERGIC53				A gift from H. Haur[7]
ss-Strep-KDEL_ss-SBP-GFP-E-cadherin				A gift from F. Perez[8]
Ii-Strep_TNFα-SBP-GFP				A gift from F.Perez[8]
ss-Strep-KDEL_SBP-mCherry-GPI				A gift from F. Perez[8]
Ii-Strep_ManII-SBP-EGFP				A gift from F. Perez[8]
DMyc-SNX3				A gift from W. Hong[9]
mCherry-P4M				Addgene #51471, a gift from T. Balla[3]
ss-GFP-KDEL				A gift from J. Lippincott-Schwartz[10]
GFP-GCC185				A gift from W. Hong[11]
HA-BIG1				A gift from M. Vaughan[12]
CD8a-furin				Previous study[1]
CD8a-sortilin				Previous study[1]
CD8a-CI-M6PR				Previous study[1]
Sec31a-mCherry				A gift from W. Hong.
mCherry-Rab5				A gift from T. Kirchhausen.

Supplementary References

1. Mahajan, D., B.K. Boh, Y. Zhou, L. Chen, T.C. Cornvik, W. Hong, and L. Lu. 2013. Mammalian Mon2/Ysl2 regulates endosome-to-Golgi trafficking but possesses no guanine nucleotide exchange activity toward Arl1 GTPase. Sci Rep. 3:3362.
2. Habib Horchani, Maud de Saint-Jean, Hélène Barelli , Bruno Antonny 2014. Interaction of the Spo20 Membrane-Sensor Motif with Phosphatidic Acid and Other Anionic Lipids, and Influence of the Membrane Environment. Plos One e113484
3. Hammond GR, Machner MP, Balla T. 2014. A novel probe for phosphatidylinositol 4-phosphate reveals multiple pools beyond the Golgi. J Cell Biol. 10.1083
4. Hammond, G. R. V., Fischer, M. J., Anderson, K. E., Holdich, J., Koteci, A., Balla, T., & Irvine, R. F. 2012. PI4P And PI(4,5)P2 Are Essential But Independent Lipid Determinants Of Membrane Identity. Science, 337(6095):727-730.
5. Lu L, Tai G, Wu M, Song H, Hong W. 2006. Multilayer interactions determine the Golgi localization of GRIP golgins. Traffic 7:1399–1407.
6. Presley JF, Cole NB, Schroer TA, Hirschberg K, Zaal KJ, Lippincott-Schwartz J 1997. ER-to-Golgi transport visualized in living cells. Nature 389:81–85.
7. Ben-Tekaya H, Miura K, Pepperkok R, Hauri HP 2005. Live imaging of bidirectional traffic from the ERGIC. J Cell Sci. 118:357–367.
8. Boncompain G, Divoux S, Gareil N, de Forges H, Lescure A, Latreche L, Mercanti V, Jollivet F, Raposo G, Perez F 2012. Synchronization of secretory protein traffic in populations of cells. Nat Methods. 9:493–498.
9. Lu, L., and W. Hong. 2003. Interaction of Arl1-GTP with GRIP domains recruits autoantigens Golgin-97 and Golgin-245/p230 onto the Golgi. Molecular biology of the cell. 14:3767-3781.
10. Ostlund C, Ellenberg J, Hallberg E, Lippincott-Schwartz J, Worman HJ 1990. Intracellular trafficking of emerin, the Emery-Dreifuss muscular dystrophy protein. J Cell Sci. 112:1709-19
11. Luke, M. R., Kjer-Nielsen, L., Brown, D. L., Stow, J. L. and Gleeson, P. A. 2003. GRIP domain-mediated targeting of two new coiled-coil proteins, GCC88 and GCC185, to subcompartments of the trans-Golgi network. J. Biol. Chem. 278, 4216-4226.
12. X. Shen, M.S. Hong, J. Moss, M. Vaughan 2007. BIG1, a brefeldin A-inhibited guanine nucleotide-exchange protein, is required for correct glycosylation and function of integrin beta1. Proc Natl Acad Sci U S A, 104:1230-1235
