# Supplementary material for: Dopey1-Mon2 complex binds to dual-lipids and recruits kinesin-1 for membrane trafficking
Source: Nat Commun. 2019 Jul 19;10:3218. doi: 10.1038/s41467-019-11056-5 (PMC6642134; doi:10.1038/s41467-019-11056-5)
Supplement: Supplementary file 2 — Description of Additional Supplementary Files [file 41467_2019_11056_MOESM2_ESM.docx]

**Description of Additional Supplementary Files**

**Supplementary Movie 1**

**Live cell imaging of peroxisomes with artificially tethered Dopey1ΔDEC.**

This movie corresponds to Supplementary Fig. 5f. Rapamycin was added at 0 min. green, GFP-Dopey1ΔDEC-FRB; red, PEX3-mRFP-FKBP. Frame interval, 60 sec; scale bar, 10 µm.

**Supplementary Movie 2**

**Live cell imaging of peroxisomes with artificially tethered DEN of Dopey1.**

This movie corresponds to Supplementary Fig. 5j. Rapamycin was added at 0 min. green, GFP-DEN-FRB; red, PEX3-mRFP-FKBP. Frame interval, 300 ms; scale bar, 10 µm.

**Supplementary Movie 3**

**Live cell imaging showing the dynamics of Golgi-to-PM trafficking carriers containing SBP-GFP-E-cadherin in a control knockdown cell.**

This movie demonstrates an example of the control knockdown panel in Fig. 7f-j. Time lapse images were acquired after warming up the system to 37 °C for 10 min. Frame interval, 350 ms; scale bar, 10 µm.

**Supplementary Movie 4**

**Live cell imaging showing the dynamics of the ER-to-Golgi trafficking of membrane carriers containing ManII-SBP-GFP in a control knockdown cell.**

This movie demonstrates an example of the control knockdown panel in Fig. 8f-j. Time lapse images were acquired after the administration of biotin. Frame interval, 350 ms; scale bar, 10 µm.

**Supplementary Movie 5**

**Live cell imaging showing FRAP of the ER pool of GFP-ERGIC53 in a control knockdown cell.**

This movie demonstrates an example of the control knockdown panel in Fig. 8k, l. Time lapse images were acquired under a spinning disk confocal microscope. The photo-bleaching was conducted at 0 min. Frame interval, 60 sec; scale bar, 10 µm.
